# Nationwide rollout reveals efficacy of epidemic control through digital contact tracing

**DOI:** 10.1038/s41467-021-26144-8

**Published:** 2021-10-11

**Authors:** Ahmed Elmokashfi, Joakim Sundnes, Amund Kvalbein, Valeriya Naumova, Sven-Arne Reinemo, Per Magne Florvaag, Håkon Kvale Stensland, Olav Lysne

**Affiliations:** 1 Simula Metropolitan Center for Digital Engineering, Oslo, Norway; 2 Simula Research Laboratory, Lysaker, Norway; 3 Institutt for informatikk, University of Oslo, Oslo, Norway; 4 Oslo Metropolitan University, Oslo, Norway

**Keywords:** Computational models, SARS-CoV-2, Epidemiology, Computer science

## Abstract

Fuelled by epidemiological studies of SARS-CoV-2, contact tracing by mobile phones has been put to use in many countries. Over a year into the pandemic, we lack conclusive evidence on its effectiveness. To address this gap, we used a unique real world contact data set, collected during the rollout of the first Norwegian contact tracing app in the Spring of 2020. Our dataset involves millions of contacts between 12.5% of the adult population, which enabled us to measure the real-world app performance. The technological tracing efficacy was measured at 80%, and we estimated that at least 11.0% of the discovered close contacts could not have been identified by manual contact tracing. Our results also indicated that digital contact tracing can flag individuals with excessive contacts, which can help contain superspreading related outbreaks. The overall effectiveness of digital tracing depends strongly on app uptake, but significant impact can be achieved for moderate uptake numbers. Used as a supplement to manual tracing and other measures, digital tracing can be instrumental in controlling the pandemic. Our findings can thus help informing public health policies in the coming months.

## Abstract (web summary)

The effectiveness of digital contact tracing for COVID-19 control remains uncertain. Here, the authors use data from the Smittestopp app, used in Norway in spring 2020, and estimate that 80% of nearby devices were detected by the app, and at least 11% of close contacts were not visible to manual contact tracing.

## Introduction

**Fig. 1:**
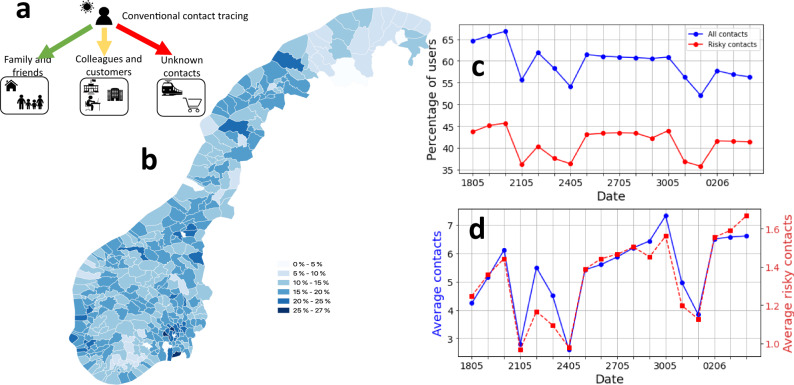
Tracking the rollout of Smittestopp and the potential for identifying risky contacts. **a** Typical settings of social contacts, the colours of the arrows capture whether manual contact tracing can succeed in identifying the contacts in the respective setting (green means complete identification, yellow partial identification, while red means zero or minor identification), **b** The percentage of population over 16 year old that were using Smittestopp in each municipality, **c** the percentage of active users per day as well as the percentage of active users that were involved in risky close contacts (within 2 metres) that lasted 15 min or more, **d** The average number of contacts per day over time, all contacts (blue) and risky close contacts (red).

When the SARS-CoV-2 virus started spreading globally, many initiatives for the development of digital contact tracing based on mobile phones were launched^1,2^. The efforts were motivated by a study by Ferretti et al., which suggested that an effective widely adopted digital contact tracing system may be enough to keep the reproduction number below 1^3^. Over a year into the pandemic, we have still not seen conclusive evidence that digital contact tracing can play a significant role in containing the pandemic. As a result, several studies have questioned the efficacy and need for digital contact tracing, especially when considering its encroachment on privacy^4–10^. Measuring the effect of digital contact tracing has been notoriously hard, as there is no contacts dataset available from a full scale production system. Further, most of the deployed systems are based on the Exposure Notification System (ENS)^11^, which is designed to greatly limit visibility into contact events in order to preserve privacy. Hence, current assessments of ENS-based apps have resorted to using a combination of incomplete data that ENS provides and population surveys^12–14^. To tackle these limitations, we used a unique real world contact data set, that was collected and anonymized during the rollout of the first Norwegian contact tracing app (Smittestopp) in the Spring of 2020^15^. Our dataset involves millions of contacts and enabled us to measure the real-world technological tracing efficacy of the app, apply a machine learning classifier to estimate the number of contacts not identifiable by manual contact tracing (see Fig. 1a) and to parameterize a model that relates tracing efficacy to the app uptake in the population. Finally, in order to assess the potential impact as a control measure, we used our efficacy estimates as an input to an established model of pandemic spreading.

We measured a high success rate in accurately detecting nearby devices (80%). Further, we estimated that a non-trivial percentage of the traced close contacts were not visible to manual contact tracing (at least 11%). We also found that the overall effectiveness of digital tracing is strongly dependent on app uptake. While an overall tracing efficacy comparable to manual contact tracing requires app uptake in the range from 80% to 90%, we found that a significant impact can be achieved for much lower uptake numbers. For example, an uptake of 40% would be enough, assuming a fast and effective case isolation, for controlling a pandemic with reproduction number of 1.5. While this study does not link digital contact tracing results with infection data, the estimated detection accuracy can serve as a reasonable proxy for the utility of digital contact tracing. Our results strengthen and add to the emerging evidence that apps are already a valuable public health tool^12–14^.

## Results

Smittestopp was rolled out in the Spring of 2020 and was quickly installed by 28% of the adult population (see Fig. 1b). The app was eventually suspended in June, because of a combination of low infection rates and privacy concerns^16^. An ENS-based app was subsequently launched in December 2020^17^. Smittestopp used Bluetooth low energy (BLE) to discover phones in a range of 10 metres. Upon a discovery event, the app measured the power of the received BLE signal, which was used to approximate the distance to the discovered device. The devices would upload their measurements to a central server, which fused the received data for identifying contacts. This centralization ensured a symmetric contact identification, since Smittestopp was asymmetric by design, that is a detection event in one direction does not imply the opposite is true.

To track the effectiveness of Smittestopp, we used anonymized daily aggregates of BLE discovery events, contacts, spanning 18 days (see Supplementary Note 2). We recorded over 26 millions contacts between 545354 phones (i.e., 12.5% of the adult population in Norway). The percentage of daily active users fluctuated between 50% and 70%. Two-thirds of the daily active users were involved in a single risky contact (see Fig. 1c), which corresponds to being within 2 metres from another person for 15 min or longer^18^. We also found that 80% of the active users had contacts on at least five different days. When considering the entire data set, we found that 89.6% of users had at least a single risky contact that lasted 15 min or longer. The observed retainability of the app and the pervasiveness of close contacts suggest a reasonable case coverage, that is the fraction of positive cases using the app. Assuming a homogeneous uniform mixing between app users and the rest of the population, we expect a case coverage close to the app adoption level.

To investigate whether Smittestopp captured movement patterns in the society, we examined the number of contacts and risky contacts over time (see Fig. 1d). The trend of risky contacts closely followed that of all contacts, and both numbers exhibited a slightly increasing trend, which is consistent with the fact that society was slowly opening up during this period. The average number of contacts dropped in weekends and national holidays, which matches the previous studies^19^.

### Estimating the technological efficacy

**Fig. 2:**
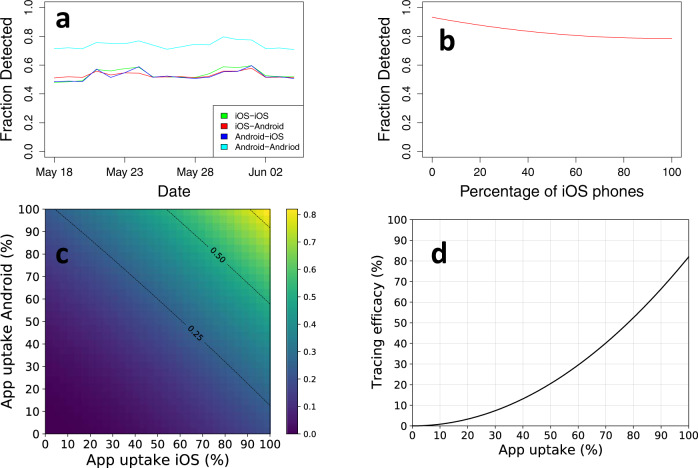
The technological efficacy of Smittestopp. **a** The probabilities of detection between different pairs of architectures as it developed over a period of 18 days, **b** The detection of contacts varies between 93% with only Android phones in the population to 79% with only iOS phones in the population. The detection rate is 80% when we have an equal split, **c** The efficacy of tracing as a function of app uptake in the two user groups. The lines mark efficacies of 25%, 50%, and 75%, respectively, **d** Tracing efficacy as a function of app uptake, assuming the same uptake in the two groups as well as an equal market share ${\phi }=0.5$.

We used the collected contact events to estimate the tracing efficacy of Smittestopp, the probability that a physical proximity event between two phones is detected by the app, and how it is impacted by app uptake. The key assumption to computing the efficacy of Smittestopp is that all phones detect each other independently. The global mobile phone market is dominated by two operating systems; iOS and Android, which we have found to differ significantly in their ability to detect contacts. We let *T* be the set of all unique ordered pairs of phone architectures, and instantiate our model by letting *T* = {*ii*, *ai*, *ia*, *aa*} where *i* means iOS, and *a* means Android. Using the collected contact events, we estimated the following probabilities: *p*_*ii*_ = 0.54 (iPhone detects iPhone), *p*_*ai*_ = 0.53 (Android detects iPhone), *p*_*ia*_ = 0.53 (iOS detects Android), and *p*_*aa*_ = 0.74 (Android detects Android). These probabilities remained stable throughout the measurement period (see Fig. 2a). The details of the underlying assumptions and calculations are provided in the Methods section.

Assuming a full app uptake in the population, the tracing efficacy of a centralized architecture, *E*, can be formulated as

1 $$E={c}_{ii}(2{p}_{ii}-{p}_{ii}^{2})+({c}_{ia}+{c}_{ai})({p}_{ia}+{p}_{ai}-{p}_{ia}{p}_{ai})+{c}_{aa}(2{p}_{aa}-{p}_{aa}^{2}).$$

Here, *c*_*z*_ denotes the probability that a physical contact between two phones is of type *z* ∈ {*aa*, *ii*, *ai*, *ia*}. The four values for *c*_*z*_ can be calculated directly from the fraction of the different operating systems of the phones using the app. If we define *M*_*i*_ = *ϕ* as the proportion of apps running on iOS phones, and *M*_*a*_ = 1 − *ϕ* as the proportion running on Android, we have

2 $${c}_{ii}={\phi }^{2},{c}_{ia}={c}_{ai}=\phi (1-\phi ),{c}_{aa}={(1-\phi )}^{2}.$$

These calculations show that the efficacy of the system depends on the distribution of iOS and Android phones in the population. The theoretical efficacy of the system in terms of detection of contacts varies between 93% with only Android phones in the population to 79% with only iOS phones in the population, as illustrated in Fig. 2b. We also found that the efficacy is dependent on the minimum duration of contacts. This is clearly evident when considering ephemeral contacts. However, we did not measure a tangible difference in efficacy when setting the minimum contact duration to 5 min as oppose to setting it to 15 min (See Supplementary Fig. 3). This is promising since it indicates a high efficacy in detecting contacts that last five minutes or longer.

In reality the uptake of contact tracing apps is well below 100%, and for Smittestopp we also observed that the uptake differed significantly between iOS and Android users. To give a realistic estimate of the app tracing efficacy, we needed to modify (1) to incorporate these factors. We define *α*_*i*_, *α*_*a*_ as the app uptake among iOS and Android users, respectively. Then (1) still holds, but with modified values for the probabilities *c*_*x*_, *x* ∈ {*aa*, *ii*, *ai*, *ia*}:

3 $${c}_{ii}={\alpha }_{i}^{2}{\phi }^{2},{c}_{ia}={c}_{ai}={\alpha }_{i}\phi {\alpha }_{a}(1-\phi ),{c}_{aa}={\alpha }_{a}^{2}{(1-\phi )}^{2}.$$

Figure 2c shows the tracing efficacy as a function of app uptake among the iOS and Android users, calculated using these modified *c*_*x*_ values with (1) and the detection probabilities *p*_*x*_, while Fig. 2d shows the tracing efficacy assuming an equal uptake by the two groups. The overall effectiveness of digital tracing is strongly dependent on app uptake, and follows the expected quadratic curve. The provided expressions for technological efficacy can be applied to systems other than Smittestopp given that the terms in Eq. (1) can be estimated. Our formulation of the technological efficacy gives an estimate of false negatives produced by the system, but it does not capture false positives. Given the modest secondary attack rate of the virus^20,21^, we expect technological false positives to have a minimal impact on the number of wrongly isolated cases (see Supplementary Note 3).

One weakness of our dataset is that it was collected from an app that was developed before the ENS, the current de-facto standard for digital contact tracing, became available. It must be noted that although ENS is expected to perform better than Smittestopp, this has not been possible to verify in any deployed system. The technical reasons for this are presented in Supplementary Information (see Supplementary Note 7). Limited experiments in controlled environments do, however, support the assumption that ENS will have an efficacy comparably to or better than we have observed in the deployment of Smittestopp^22–24^, and thereby support our conclusions on the potential of digital contact tracing. The available follow-up data on deployed ENS-based apps is limited. We used publicly available statistics about the German and Swiss official apps to gauge their efficacy^25,26^. These two apps were rolled out in June 2020. Inline with our results from Smittestopp, the app uptake seems to be a good proxy for gauging the case coverage (see Supplementary Note 7). We also note that apps like Smittestopp can potentially run on any phone with BLE support. Prior to December 2020, ENS was supported on iPhone devices that were released after 2015. A recent update extended the support to older phones making ENS available on most phones that were released after 2012 (more details are available at https://covid19.apple.com/contacttracing).

### Detecting unknown close contacts

To check whether Smittestopp was successful in detecting untraceable close contacts, we built a machine learning classifier to separate unknown (random) contacts from known close contacts (see the Methods). The model learned association patterns from the contacts graph and achieved an accuracy of 89% when classifying risky close contacts. Overall, at least 11% of the risky close contacts were random.

**Fig. 3:**
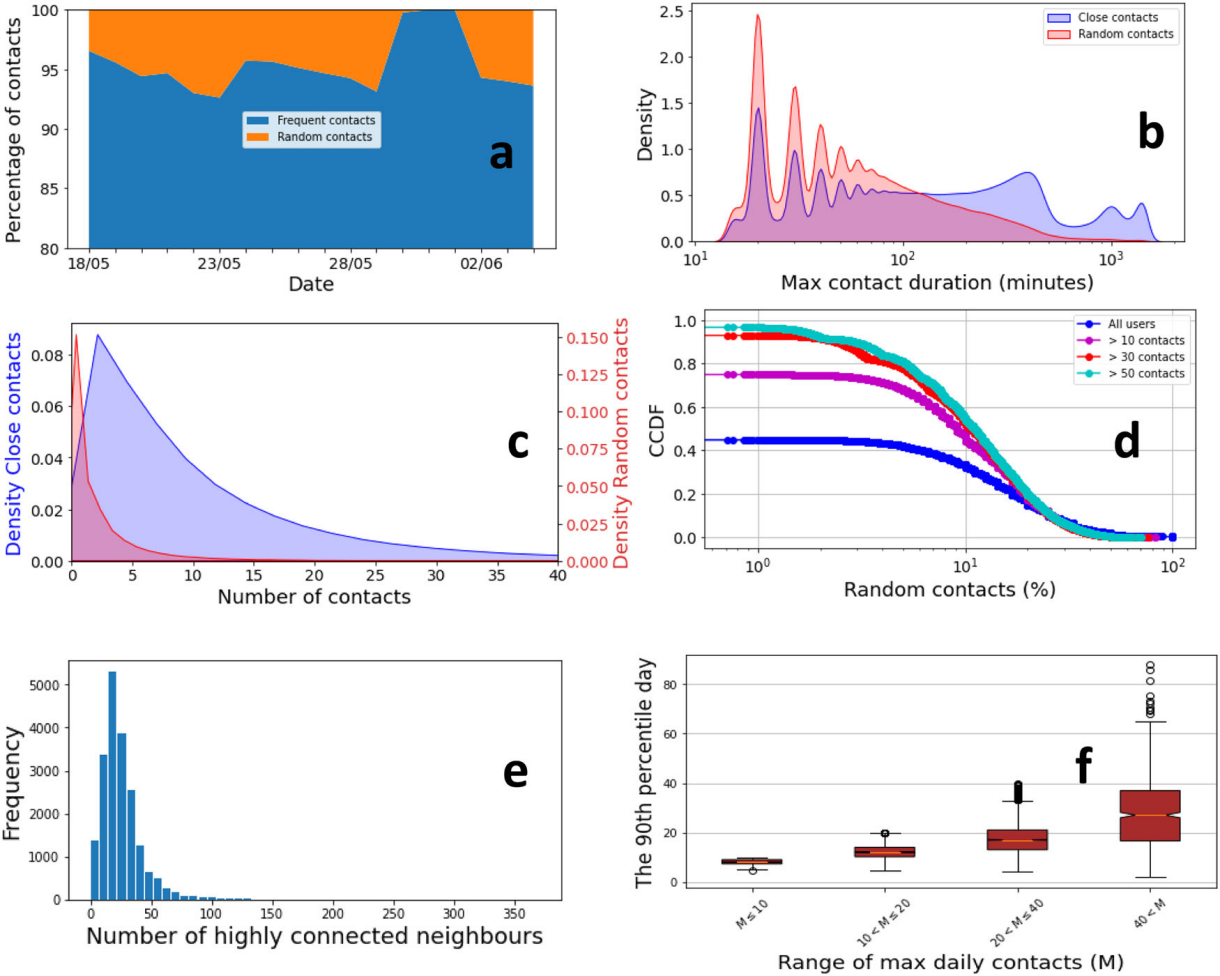
Quantifying the success in detecting unknown close contacts. **a** The contacts split over time. The fraction of risky random contacts is stable over time and decreases in weekends and holidays, **b** The density of encounter duration. Frequent contacts tend to be markedly longer, **c** The density of the number of contacts per user, which are split based on the inferred contact type, **d** The Complementary Cumulative Distribution Function (CCDF) of the percentage of random contacts per user for all users, users with 10 contacts or more (49% of all users), users with 30 contacts or more (12% of all users) and users with 50 contacts or more (4% of all users), **e** The histogram of the number of highly connected neighbours per a highly connected user. A user is highly connected if he/she has 50 or more contacts, **f** The distribution of the 90^*th*^ percentile day in terms of number of daily contacts for highly connected users. Here we grouped highly connected users based on the maximum number of daily contacts per user. In the box-whisker plot, the central line indicates median, box limits indicate upper and lower quartiles, and whiskers specify 1.5 times the interquartile range above the upper quartile and below the lower quartile. The circles above and below the whiskers indicate the samples that are either larger or smaller than the whiskers. These are the outliers in each range. The number of samples *N* is 794 for *M* ≤ 10, 9715 for 10 < *M* ≤ 20, 8035 for 20 < *M* ≤ 40 and 876 40 ≤ *M*.

The fraction of daily random contacts varied slightly over time, but remained around 6% (see Fig. 3a). It dropped during holidays, notably the long Ascension day weekend at the end of May, and it peaked in the days leading to the holidays. We performed the same analysis as we varied the threshold for considering a contact as risky. The fraction of risky random contacts increased to 33.3% when abolishing the duration threshold. We, however, note that our estimates of random contacts are conservative (see the Methods and Supplementary Note 5). Random contacts were shorter, mostly lasting between 20 and 40 min (see Fig. 3b). This is, however, a duration long enough to spread infection. Close contacts were longer and can last a whole day (i.e., household contacts) or several hours with a peak around 7 hours (i.e., work contacts). The number of random contacts per user, in the entire study period, was far less than close contacts. Over half of the users did not have random contacts (i.e., only known contacts), but over 30% of the users had 10% or more random contacts (see Fig. 3c, d). The lack of random contacts, for over 50% of the users, can be related to the imposed lockdown and adherence to social distancing as well as our conservative estimates.

Considering only users with a relatively high number of contacts, the percentage of users with 10% random contacts increased to between 50% and 60%. Overall, the top 20% of users, in terms of contacts, had 20% or more random contacts. To gain insight into the nature of these active users, we took a closer look at the users with 50 contacts or more, which we refer to in the following as highly connected users. We first examined the number of highly connected neighbour per such user (see Fig. 3e). The majority of highly connected users have a non-trivial number of highly connected neighbours. In fact, only 7% of them do not connect to any other highly connected users. The distribution exhibits a pronounced mode at 14 highly connected neighbours, this mode describes 27% of the users. There is, however, a small fraction of users, about 4%, that are connected to more than 59 other highly connected users. This high linkage likelihood, amongst highly connected users, suggest that such users may belong to a work environment that exposes them to excessive close contacts, e.g., health personnel, shop keepers, rail conductors^27^. They can also be individuals that were involved in superspreading events. Tracking the distribution of the number of daily contacts that a highly connected user has can shed light on the mechanism underlying the high connectedness. For instance, users with a high number of contacts over several days are probably part of a work environment with frequent social encounters. To this end, we divided the highly connected users into buckets based on the maximum number of daily contacts they had, then examined the distribution of the 90th percentile day within each bucket. For most users, there is a reasonable agreement, in the number of contacts, between the 90th percentile and maximum days (see Fig. 3d). In other words, these users were highly connected on more than a single day. We also observed a small fraction of users with close to zero contacts in most days. Our observations suggest that a non-trivial fraction of highly connected users have an occupation that exposes them to excessive close contacts, e.g., health personnel, shop keepers, rail conductors^27^. Further, we also observed users that were highly connected on only a single day. These findings were further confirmed when breaking down encounters involving highly connected users by day of the week as well as when examining the tendency of highly connected users to run into other highly connected users (see Supplementary Fig. 7 and Supplementary Fig. 8). This analysis indicates that digital contact tracing can potentially help containing outbreaks that are related to highly connected individuals and superspreading events.

The detected fraction of random contacts suggest that the app can significantly supplement manual contact tracing. If we assume, for instance, 60% app uptake in the population, we observe from Fig. 2d that the efficacy of the app tracing is approximately 30%. This app will improve the overall tracing accuracy by 7.5–10.5% in a society where the fraction of random contacts is between 25% and 35%, respectively. Here, we assume that manual contact tracing identifies all non-random contacts. A supplement of this magnitude can mean the difference between a controlled pandemic and an exponential growth of cases^3^.

### Effect on spread of SARS-CoV-2

**Fig. 4:**
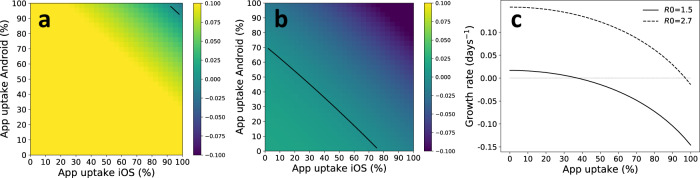
The impact of app uptake on the spread of SARS-CoV-2. The plots show the estimated growth rate *r* as a function of app uptake among Android and iOS users. We have assumed 90% efficacy of case isolation and a 4 h delay of both case isolation and contact quarantining. **a** Shows the situation for R0 = 2.7 and **b** shows R0 = 1.5. **c** Shows the same data, but assuming identical app uptake among iOS and Android users. Note that R0 denotes the initial reproduction number of the pandemic, which is the expected number of new cases that are caused by an infected individual.

After investigating the technological efficacy and its viability in detecting contacts, we turned to assess its potential impact on the pandemic spread. The tracing efficacy as a function of app uptake was computed from (1), as illustrated in Fig. 2c, and then these numbers were input to the model of Ferretti et al^3^, which describes the effect of contact tracing on pandemic spread. Figure 4a, b shows the estimated growth rate *r* (in days^−1^), as a function of app uptake in the two user groups. In Fig. 4a, we chose the initial reproduction number as R0 = 2.7, which is in line with reported numbers from the early phase of epidemic spread in various countries^28,29^. Figure 4b shows the growth rate for R0 = 1.5, chosen to represent a more slowly growing epidemic resulting from control measures such as social distancing. In both plots, the efficacy of isolating symptomatic cases was set to 70%, we assumed a four hour delay in both case isolation and quarantining of contacts, and the proportion of environmentally transmitted (i.e., non-traceable) infections was set to 10% inline with our estimates. Results for other parameter choices are included in Supplementary Note 9. The black lines show *r* = 0, i.e., the threshold between increasing and declining numbers of infected cases. Figure 4c shows the same results as curve plots, assuming identical app uptake in the two user groups.

Figure 4a indicates that controlling the pandemic using app-based contact tracing alone is probably unrealistic, since 95% of the population would need to install the app to control a pandemic with an initial reproduction number R0 = 2.7. However, the situation is completely different for the case of R0 = 1.5, which may be more representative of a situation with other controlling measures in place. In this case, the required uptake was about 40% to control the epidemic and achieve a decline in the number of cases. Unfortunately, the majority of countries struggle with pushing app uptake beyond 20–25%. However, a few countries including the UK and Denmark, provide a cause for optimism, by reaching a 30% or more uptake rate. For example, 49% of the eligible population with compatible phones have installed the NHS app in England and Wales^14^.

## Discussion

Our analysis of the data from the first rollout of Smittestopp reveals some central findings. The first is that it is possible to reach a significant efficacy of digital contact tracing on mobile phones. With an equal split between Android and iOS-phones in the population, we measured an efficacy above 80%. Although many discussions on the topic have taken this for granted, it should be noted that this was far from obvious. The phone-models used in a given society varies enormously, and none of the models were designed with contact tracing in mind. Documentation of an efficacy of above 80% in a rolled out solution is therefore a decisive finding. We expect ENS-based apps to achieve a comparable or better accuracy, given their superiority to Smittestopp. The second finding is that when used in a real population, a digital contact tracing system does detect a non-trivial number of close contacts that is out of reach for manual contact tracing. Our machine learning model concluded that at least 11% of the contacts with high risk of infection spread were random, and would likely not have been identified with manual contact tracing. The third finding is that measurements from a full scale rollout combined with epidemiological models show significant potential contributions from digital contact tracing to stopping the pandemic. Although there is significant room for improvement of technical accuracy, it appears that the app uptake rate in the population is the only real impediment for realizing the potential of digital contact tracing. Yet, tangible benefits are possible at modest uptake rates. An uptake rate of 40%, for example, can help reducing the reproduction number in the current phase of the pandemic, where the reproduction number is between 1 and 1.5 in most countries^30,31^. This study does not link the traced contacts with infection data, which limits our ability to gauge the actual impact of digital contact tracing on slowing down the pandemic. We, however, believe that the technological efficacy can serve as a relevant proxy in this context.

With Covid-19 expected to be endemic^32^, new virus variants^33^ and a promising but lengthy vaccination campaign^34^, our findings suggest that digital contact tracing can greatly boost efforts to control the pandemic in the coming months. Health authorities should have an immediate focus on increasing the uptake of contact tracing apps. This should be supplemented with efforts to establish digital contact tracing as an essential tool for public health.

## Methods

### Modelling the technological efficacy

A central assumption in the model is that the phones detect each other completely independently. This means that whenever phone *A* detects phone *B*, this detection event does not change the behaviour of phone *B* in a way that will affect its probability of detecting phone *A*. There is nothing in the implementation of Smittestopp that should imply that this assumption does not hold. The code was written such that the act of detecting another phone, and the act of being detected by another phone are not dependent on each other.

Assume two types of phones, *x* and *y*. When two such phones are in proximity of each other, let *p*_*xy*_ be the probability that *x* detects *y*, and *p*_*yx*_ be the probability that *y* detects *x*. Let *C*_*xy*_ be the number of factual proximity events, i.e., contacts, over a given period, between two app users carrying phone of type *x* and *y*, respectively. Furthermore, let *D*_*xy*_ and *D*_*yx*_ be the number of these events that are detected by the phone of type *x* and *y*, respectively, and let *D*_*xy*+*yx*_ be the number of proximity events detected by both phones.

Then the following equations hold:

$${C}_{xy}{p}_{xy}={D}_{xy},$$

$${C}_{xy}{p}_{yx}={D}_{yx},$$

$${C}_{xy}{p}_{xy}{p}_{yx}={D}_{xy+yx}.$$

Solving these equations for *p*_*xy*_ and *p*_*yx*_ gives

4 $${p}_{xy}=\frac{{D}_{xy+yx}}{{D}_{yx}},$$

5 $${p}_{yx}=\frac{{D}_{xy+yx}}{{D}_{xy}}.$$

Note that these equations are valid regardless of the number of types of phones there exist, and they also hold if *x* and *y* are identical.

If we now let *T* be the set of all unique ordered pairs of phone architectures, we can formulate the tracing efficacy *E* of a centralized architecture as

$$E=\mathop{\sum}\limits_{xy\in T}{E}_{xy}$$

where

$${E}_{xy}={c}_{xy}({p}_{xy}+{p}_{yx}-{p}_{xy}{p}_{yx}).$$

Here *c*_*z*_ denotes the probability that any given contact between two phones is of type *z* ∈ {*xx*, *yy*, *xy*, *yx*}. Note that in this formulation, *c*_*xy*_ = *c*_*yx*_, whereas *p*_*xy*_ and *p*_*yx*_ are in general different, due to technological differences between the phones. The values for *c*_*z*_ can be calculated directly from the fraction of the different types of phones using the app.

The mobile phone market in the world is dominated by two operating systems; iOS and Android, with significantly different properties. There is a rich set of different phone models as well, but the differences between the operating systems dominate the picture. We therefore instantiate our model by letting *T* = {*ii*, *ia*, *ai*, *aa*} where *i* means iOS, and *a* means Android, which yields the total tracing efficacy defined in Eqs. (1)-(2) above.

Note that the formulation in (1) mandates that data is collected centrally. If two phones are in proximity of each other, it suffices that the contact is detected by at least one of them. Our results show that this property of Smittestopp dramatically improved the efficacy of the system, although at the cost of higher risks to privacy. The technology developed by Apple and Google, which was made available after the launch of Smittestopp, eventually made this trade-off between efficacy and privacy less critical. Note also that (1)-(2) define *E* as the ratio of detected contacts to the total number of actual contacts among app users. We have ∑_*x*_*c*_*x*_ = 1, and in a situation with 100% app uptake in the population, *E* would be the total efficacy of the system in detecting contacts. In reality the app uptake is well below 100%, and contacts will also occur between phones without the app installed. To describe the total tracing efficacy in this situation we introduce the modified contact probabilities in (3), where we have defined *α*_*i*_, *α*_*a*_ as the app uptake among iPhone and Android users, respectively. Here we have ∑_*x*_*c*_*x*_ < 1, since we only capture contacts where both phones have the app installed. Eq. (1) still holds for the total efficacy, but with the contact probabilities given by (3).

We used the contact dataset (see Supplementary Note 2 for details on the dataset) to calculate the number of detected contacts *D*_*ia*_, *D*_*ai*_, and *D*_*ia*+*ai*_, and from (4)-(5) we got the following probabilities:

- Probability that iOS detects iOS; *p*_*ii*_ = 0.54
- Probability that Android detects iOS; *p*_*ai*_ = 0.53
- Probability that iOS detects Android; *p*_*ia*_ = 0.53
- Probability that Android detects Android; *p*_*aa*_ = 0.74

More specifically, we focused on the most relevant contacts from an epidemiological point of view (i.e., within 2 metres and lasting at least 15 min)^18,35^. The numbers of detected contacts *D*_*ia*_, *D*_*ai*_, and *D*_*ia*+*ai*_ were computed directly since each contact is associated with a direction and labelled with phone types (i.e., phone *A* of type *x* detected phone *B* of type *y*).

### Identifying random contacts

We used a random forest binary classifier^36,37^ to separate close contacts into known and random. To train a binary classifier, we needed a training set that includes both true positives (i.e., known contacts) and true negatives (i.e., random contacts). In absence of a verified ground truth, we needed to carefully pick these two sets from the underlying data. As true positives, we picked device pairs that met on at least seven different days. We examined contact patterns to discern potential true negatives. More specifically, we picked device pairs that were never in contact, despite both being in a repeated close contact with a common third device, as true negatives (see Supplementary Note 5 for more details).

We trained a random forest classifier with 20 trees, gini criterion, a maximum tree depth of 8, a minimum number of samples required to split an internal node of 2 and a minimum number of samples required to be at a leaf node of one. The values of these hyperparameters were selected after conducting an exhaustive grid search. Overall, we used nine features that were meant to capture the quality of information we have on a pair of devices, their connectivity as well as the topological commonalities between them (i.e., how many neighbours they share). We fitted three models for close contacts of any duration, at least five-minute long and at least fifteen-minute long. The three models exhibited an accuracy of 84%, 88% and 89%, respectively. We classified between 11% and 33.3% of contacts as random depending on the definition of close contact. Our model can classify contacts between devices with at least a single common neighbour. One-off contacts between devices without common neighbours could not be classified and were assumed to be known close contacts in order not to inflate the added value of digital contact tracing. Hence, our estimates of the fraction random contacts are conservative. A detailed analysis of these aspects is provided in Supplementary Note 5.

### Ethical considerations

The privacy considerations of Smittestopp was subject to a fierce public debate, thus any use of data from this app has to be done with great care. Both the legality of data usage for this particular study, and the potential damage that the existence of the dataset could lead to was considered by the management of Simula Research Laboratory before they authorised the study.

Simula received the necessary consent from the Norwegian Institute of Public Health to use the data-set for research purposes, which is in accordance with the privacy policy of Smittestopp. Adding to this, Simula collected separate legal assessments from the Norwegian Centre for Research Data (NSD) on the risk of re-identification based on our data-set, and from the Norwegian Law-firm Wiersholm on the legality of the data-set. Based on the received advice, Simula concluded that the use of the data-set for research purposes is legal.

Regarding the potential for damage done through the existence of the dataset, Simula leans on the received assessment from NSD that re-identification of individuals from the dataset is hard to imagine. Still, as an extra level of caution, Wiersholm suggests that Simula should not make the dataset public, but rather share it with other research institutions under agreements of non-disclosure and usage limitation. Simula’s treatment of the dataset is in line with this suggestion. More information on the legal status of the data set is given in Supplementary Note 10.

### Reporting summary

Further information on research design is available in the Nature Research Reporting Summary linked to this article.

## Supplementary information

Supplementary Information

Reporting Summary

## Data Availability

The data used for calculating the detection probabilities between different pairs of architectures in this study have been deposited in a public Github repository^38^. The contact data set is available to research institutions and for research purposes upon request to Simula Research Laboratory as an extra level of caution due to privacy concerns (please refer to Ethical consideration and Supplementary Note 10 for extra details). Requests will be authorized by Simula’s management. Correspondence and requests for materials should be addressed to Ahmed Elmokashfi or post@simula.no. The data for assessing the impact of uptake on pandemic progression (i.e., Fig. 4) and analysis of the ENS are available in the same public repository^38^.

## Code availability

The scripts for the efficacy model, the impact of uptake on pandemic progression (i.e., Figs. 2 and 4), analysis of the ENS and the analysis of random contacts and highly connected users (i.e., Fig. 3) are available at ref. ^38^. These script are developed using Python version 3.8.10, R version 4.0.3 and Perl version 5.30.0. This work is licensed under a Creative Commons Attribution 4.0 International (CC BY 4.0) license, which permits unrestricted use, distribution, and reproduction in any medium, provided the original work is properly cited. To view a copy of this license, visit https://creativecommons.org/licenses/by/4.0/. This license does not apply to figures/ photos/artwork or other content included in the article that is credited to a third party; obtain authorization from the rights holder before using such material.
