# Supplementary material for: Nationwide rollout reveals efficacy of epidemic control through digital contact tracing
Source: Nat Commun. 2021 Oct 11;12:5918. doi: 10.1038/s41467-021-26144-8 (PMC8505561; doi:10.1038/s41467-021-26144-8)
Supplement: Supplementary file 1 — Supplementary Information [file 41467_2021_26144_MOESM1_ESM.pdf]

Supplementary Material for Nationwide rollout reveals efficacy of epidemic control through digital contact tracing

Ahmed Elmokashfi[1,*], Joakim Sundnes[2], Amund Kvalbein[1], Valeriya Naumova[1], Sven-Arne Reinemo[1], Per Magne Florvaag[2], Håkon Kvale Stensland[2,3], and Olav Lysne[1,4]

[1]Simula Metropolitan Center for Digital Engineering, Oslo, Norway
[2]Simula Research Laboratory, Lysaker, Norway
[3]Institutt for informatikk, University of Oslo, Oslo, Norway
[4]Oslo Metropolitan University, Oslo, Norway
[*]Corresponding author, ahmed@simula.no

Supplementary Note 1: Smittestopp

As a response to a rising number in Covid-19 cases, the Norwegian health authorities decided on the 13th of March, 2020 to develop a contact tracing application. The app was subsequently launched on the 16th of April, which was received positively by the population[1]. The number of app downloads reached 1.5 million two weeks after the launch date. The number of active users per day (i.e. users that shared tracing information) peaked around 800k in the first few days post launch then decreased steadily to approximately 500k in early June (see Supplementary Figure 1). The phone population was dominated by iOS devices but twice as many Android devices were lost in the course of the app deployment compared to iOS. The difference in the adoption rate between the two platforms reflects their popularity. The discrepancy in lost users can be blamed on the app resulting in a higher energy consumption on Android. Optimizing for Android was particularly hard given the high diversity in terms of both vendors and devices. The active users of Smittestopp were distributed across the country with a higher concentration in major urban hubs (see Figure 1b in the main text). Smittestopp was suspended on the 16th of June due to privacy concerns and decrease in infections[2]. Norway released a new app based on the exposure notification system in December 2020[3]. The source code of Smittestopp is publicly available[4]. We note that the aggregate numbers behind Supplementary Figure 1 and

Figure 1b in the main text were collected when the app was operational. Figure 1b assigns users to municipalities based on the measured GPS coordinates at 02:00 am.

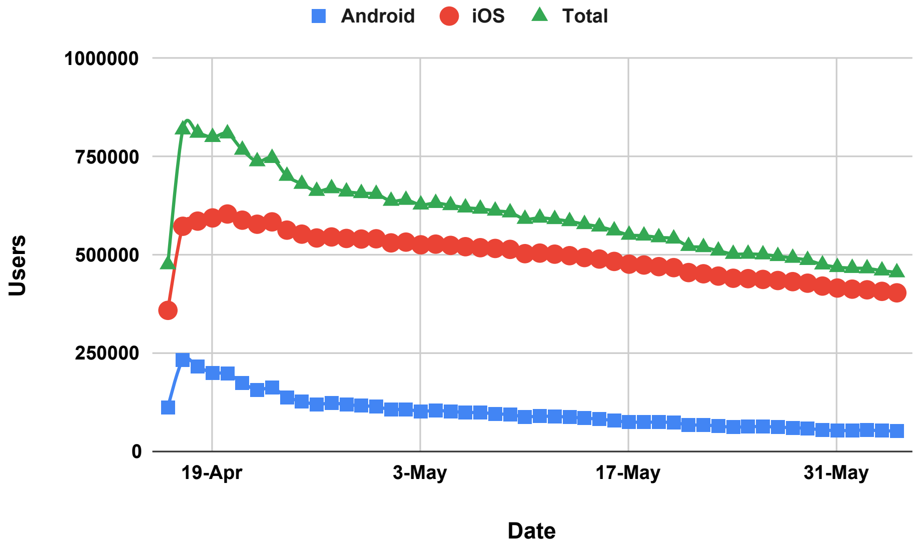

Supplementary Figure 1: The number of active users per day.

Smittestopp was intended to both automate contact tracing and collect aggregate information on the mobility and interactions between users. This information was needed to help inform government policy on pandemic control. To this end, Smittestopp logged both users GPS locations and used Bluetooth Low Energy (BLE) to discover other users in proximity [5]. Phones with Smittestopp would continuously broadcast their presence over BLE as well as periodically scan for phones with Smittestopp in proximity. Smittestopp used a universally unique service identifier for advertising presence and scanning for nearby devices. Note that BLE signals have a 10-meter propagation range. Upon the discovery of a nearby device, the phone would connect to it and measure the strength of the received BLE signal. Phone pairs acted independently meaning that the event a phone A discovering another phone B did not automatically translate to a discovery event in the opposite direction. In other words, the discovery in Smittestopp was asymmetric. Smittestopp was a centralized solution, that is all phones would upload their GPS and BLE measurments to a central database. This design choice was necessary for providing aggregate information on the mobility and interactions between users. It also transformed the discovery process from asymmetric to symmetric.

Supplementary Note 1.1: The ”iPhone-problem”

In the main text, we pointed to the fact that iOS and Android differ when it comes to the effectiveness of detecting nearby phones. The difference is related to limitations imposed by iOS on apps that run in the background, that is the app is running while the user is looking at another

app or the screen is turned off. These limitations manifested in two forms. First, Apps using BLE and running in background were suspended by iOS a few second after entering the background mode. Suspended apps would still be visible to nearby phones that were active (e.g. Android phones or iOS devices with the app in the foreground). If a suspended app was discovered by another phone, iOS would bring it for approximately 30 seconds to the background, which means it could scan for other devices for a short while. Second, iOS would alter the format of BLE advertisement packets, sent by an app in the background, to a proprietary one. This makes it harder for other devices to discover nearby phones with Smittestopp running in the background[6,7]. These limitations implied that two iPhone devices with apps in background would not be able to detect each other. In other words, Smittestopp would not report that two iPhone users sitting next to each other in a bus with phones in their pockets as a close contact. This was a major hurdle given the large fraction of Smittestopp users that had iOS devices. Note that these limitations faced all apps that were not built using the Exposure Notification System[8].

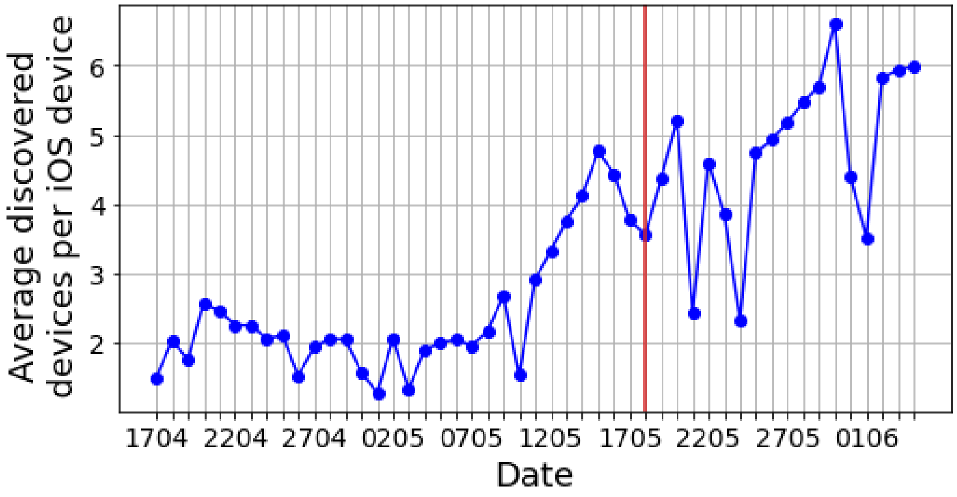

Supplementary Figure 2: The average number of detected phone by an iOS device over time.

Smittestopp team eventually found a work around that would partially handle the aforementioned limitations. The work around leveraged the iBeacons and locations framework in iOS to periodically scan for BLE beacons in proximity[9,10]. A BLE beacon is a fixed device that periodically advertises its presence to nearby phones, which is typically used in indoor settings to help in navigation to various points of interest. Smittestopp scanned for non existent beacons, a positive side effect of this was that iOS would, during the scanning, relay BLE packets to apps in the background. The only caveat was that the screen needed to be on. So, a user flipping through the news, for example, was visible to nearby phones although the app was in the background. Even brief screen on events, for example in connection with the arrival of any notification, would make the phone detectable. The work around was rolled out in early May and immediately led to an increase in the detection rate for iOS devices (see Supplementary

Figure 2). The average number of detected devices increased steadily starting on the 8th of May. It continued climbing up as more users downloaded the update and eventually stabilized around the 18th of May (the red line in Supplementary Figure 2).

Supplementary Note 2: Dataset

Our dataset comprises a summarized set of BLE measurements that was collected by Smittestopp. It is technically pseudonymized per person. Since there exists no auxiliary dataset that supports re-identification of individuals, the dataset is deemed anonymized in the legal sense of the word. Note that all auxiliary data that can be used to re-identify users was deleted (see Supplementary Note 10 for details on the legal aspects of the data set). The original data was deleted, because Smittestopp's privacy policy committed to deleting all raw data that was older than 30 days.

Data aggregation. The data was aggregated on daily basis. All device pairs that were in proximity were identified as contacts. A contact corresponds to a series of N BLE measurements, where $N \geq 1$. Every measurement i is a tuple $(t_i, RSSI_i)$, where t_i is the measurement timestamp and $RSSI_i$ is the measured BLE signal strength. All measurements in a contact, within a single day, were sorted, then the daily contact was defined as spanning the entire duration between the earliest and latest timestamps t_i and t_N, respectively. Also the maximum and average RSSIs were recorded. As a result, a daily contact between two devices p_a and p_b is a tuple of $(p_a, p_b, \Delta t, RSSI_{max}, RSSI_{avg}, N, T_a, T_b)$, where Δt is the time difference between t_N and t_i, $RSSI_{max}$ is the strongest signal strength, $RSSI_{avg}$ is the average signal strength, T_a is the type of device p_a and T_b is the type of device p_b. Note that T_a and $T_b \in (iOS, Android)$. We note that the data is not aggregated across users. Each data record corresponds to an encounter between two specific users. However, all daily measurements, per this encounter, are aggregated.

This approach to data aggregation masks all details about the time of contact, which is essential for ensuring users' privacy. This has the side effect of mischaracterizing a pair of short encounters that were spaced by several hours as a single long varying contact. For example, two unrelated individuals that sat in the same train carriage in the morning and the afternoon of the same day. Filtering on the measured signal strength reduces the impact of such false positives by discarding encounters that are associated with a weak signal. Further, if such a contact was observed over a number of days with a signal strength that indicates a close spatial proximity, we can assume that the contact may qualify as a valid close contact.

Data anonymization/pseudonymization After aggregating encounters between device pairs into daily contacts, the device identifiers (i.e. p_a and p_b above) were hashed using the Secure Hash Algorithm 2 (SHA-2) with a 256 bits digest[11]. This algorithm produces a hash that can not be traced back to the original device identifier. It always, however, maps a device identifier to the same hash, which allows to track the activity of a device across several days. After the initial hashing step, each hash was mapped to a random number in the range $(1, ..., N)$, where N is the total number of devices, then the hashes were deleted. These random numbers were generated using the default seed which is the system clock timestamp. The mapping from the hashes to the random numbers is not reversible because the original hashes were deleted. Every

contact in the dataset can not possibly be mapped to a particular individual, since the raw data was deleted. Further, the contact does not include additional spatial details like GPS coordinates or even any fine granular details on devices beyond being an iOS or Android device.

Basic contacts statistics. The dataset spans the period from the 17th of April to the 4th of June 2020, that is from the first day after Smittestopp was released to ten days before it was suspended. As explained in Supplementary Note 1.1, Smittestopp underwent a major update in early May to address the iOS-imposed limitations. This translated into a higher rate of false negatives in the first three weeks of Smittestopp's lifetime. We therefore considered mainly the data collected between the 18th of May and 4th of June, that is a total of 18 days. In this 18-day period no updates of the app were pushed to the phones, and the effects of previous updates of the app had stabilized. In order to avoid spurious users who downloaded the app and stopped using it after a short while, we removed from the dataset all devices that were not present on seven different days (i.e. the device was seen in a contact on seven different days). We also considered only contacts between devices that had a 7-day overlap, meaning that both devices appeared in the dataset on at least the same seven days.

We further derived three datasets as follows:

1. *Proximity Contacts (PC).* These are contacts of any duration but with an average RSSI that is consistent with 2 meters proximity. To convert from RSSI to distance, we used the thresholds that were set by Smittestopp[12], where contacts with $RSSI \geq -85$ dBm were considered as close. Mapping RSSI values to physical distance is known to be imprecise and challenging[13]. However, our evaluations of Smittestopp accuracy in both controlled and real-life scenarios showed high accuracy(see the tables and analysis on pages 21-23 in [12]). We plan to take a closer look at BLE accuracy and the potential for improving it by either leveraging contextual details or using novel algorithms[14].

2. *Relatively Risky Contacts (RRC).* These are contacts that were at least 5 minutes long with an average RSSI that is consistent with 2 meters proximity.

3. *Potentially Risky Contacts (PRC).* These are contacts that were at least 15 minutes long with an average RSSI that is consistent with 2 meters proximity. PRC contacts are associated with a higher covid-19 transmission risk, if one of the involved individuals is contagious.

Accordingly, $PRC \subset RRC \subset PC$. Supplementary Table. 1 summarizes the number of contacts and devices in our dataset. The number of unique devices is about 545k, which amounts to 12.5% of the Norwegian population over 16 years old. [1] The number of unique devices drops as the dataset becomes stricter. Overall, 10.8% of all contacts were *PRC*.

Supplementary Note 3: Bluetooth and False Positives

[1]Smittestopp had an age limit of 16.

Supplementary Table 1: Basic statistics of contacts. Note that contacts are divided into three types: Proximity Contacts (PC), Relatively Risky Contacts (RRC) and Potentially Risky Contacts (PRC). These types capture the duration and associated covid-19 transmission risk.

	All	PC	RRC	PRC
Unique devices	545354	525646	485730	461068
Unique contacts	26792433	10665399	3946986	2889922

Our approach for estimating the efficacy of Smittestopp does not capture the number of false positives generated by the system. We do not believe that this information is to be found in the dataset, as the dataset contains no ground truths on proximity. These false positives stem from the fact that the BLE signal can exhibit non-trivial propagation patterns depending on surrounding environments[15]. For example, environments with metallic elements like the inside of a tram can amplify BLE signal and thus underestimate the actual distance. Also phones separated by thin walls may appear closer than they really are as far as the virus spreading is concerned.

We, however, argue that the number of false positives generated by BLE is at the level of noise when compared to the false positives stemming from accidental lack of infection spread. Let us first define a false positive contributed by the technology to be a registered contact, where the definitions of what constitutes a contact does not hold. Since BLE will only communicate over short distances, usually up to 10 meters, a false positive according to this definition will amount to situations where the following holds:

- There is a proximity between the two persons of 10 meters or less. If this was not the case the phones would be out of range for each other's BLE signal, and no registration would be made.
- The proximity between the persons lasted for 15 minutes or more - otherwise it would not be registered as a contact.
- The true distance between the two persons would be more than 2 meters - otherwise it would not be a false positive.
- The measurements from BLE falsely indicate a distance of less than 2 meters for 15 minutes.

There will be situations in daily life where these four requirements hold, for example people seated within 10 meters of each other on a bus or in a theater. Still, for this to be a dominating factor, two things must be true. First, the disease must be extremely contagious, so that most contacts within 2 meters in 15 minutes get infected. Second it must be extremely accurate so that contagion stops at 2 meters, and starts after 15 minutes. This is far from being the case for Covid-19. Various studies estimate the secondary attack rate for Covid-19 at 17% for household contacts and 27.8% to contacts who were spouses of index cases[16,17]. The secondary attack rate is markedly lower for settings outside the household[18,19]. Accordingly, both manual and digital

contact tracing will yield a high fraction of false positives from an epidemiological point view, that is identified contacts that did not contract the virus.

Supplementary Note 4: The Technological Efficacy of Digital Contact Tracing

Supplementary Figures 3a and 3c show the evolution in the basic four probabilities, which we need for estimating the technological efficacy, for the PC and RRC datasets, respectively. The remaining two panels in the figure show the estimated efficacy as a function of the percentage of iPhones in the population. The probabilities improve as the observed duration of the contact increases. Interestingly, the improvement is relatively small when moving form RRC to PRC (see Fig. 2 in the main text). This small improvement results in essentially identical detection accuracy.

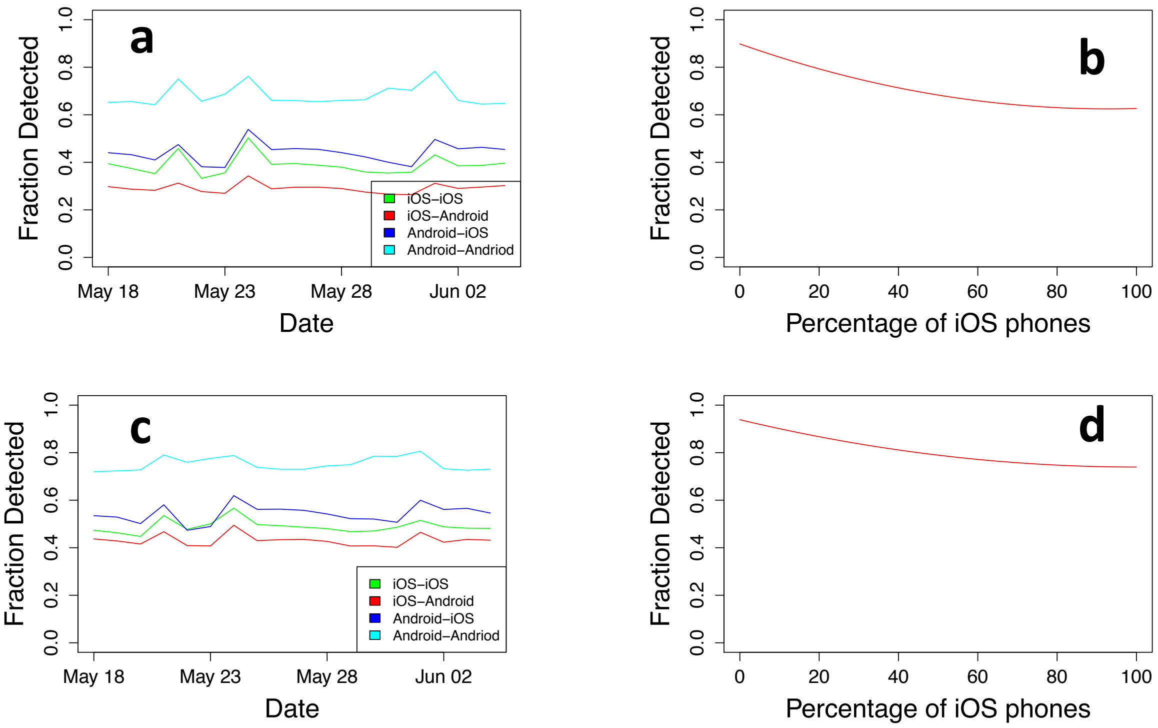

Supplementary Figure 3: **a)**: The probabilities of detection between different pairs of architectures, for PC contacts, as it developed over a period of 18 days, **b)**: The fraction of detected PC contacts as a function of the fraction of iPhones in the population, **c)**: The probabilities of detection between different pairs of architectures, for RRC contacts, as it developed over a period of 18 days, **d)**: The fraction of detected RRC contacts as a function of the fraction of iPhones in the population.

**Dataset**	% of one-off contacts
PC	77.1%
RRC	64.1%
PRC	57.3%

Supplementary Table 2: The fraction of one-off contacts per type of contacts. Recall that contacts are divided into three types: Proximity Contacts (PC), Relatively Risky Contacts (RRC) and Potentially Risky Contacts (PRC). These types capture the duration and associated covid-19 transmission risk.

Supplementary Note 5: Classifying contacts

Contacts can either be known or random. Manual contact tracing can in principle identify all known contacts given that the case recalls all recent encounters. Random contacts, however, can not be identified by manual contact tracing alone. An additional approach that tracks the presence of unrelated individuals in a particular location at a particular time, like digital contact tracing or restaurants guest lists, is needed.

Our dataset does not include any extra information, like GPS coordinates or a user-provided context, to help separating known from random contacts. Nevertheless, it tracks contacts over time as well as contact duration and these two can provide an idea about repeated long encounters. The contact dataset can also be represented as a network with devices as nodes. A pair of nodes are connected, if a contact is recorded between them. The corresponding edge weight is the number of unique days with contacts. This contact graph can give insights into similarity between devices, in terms of presence of common neighbours, which can be used in inferring known contacts.

The lack of ground truth, however, complicates the task of validating the outcome of the classification process. To address this, we leveraged the intuition that random encounters tend to be shorter than known contacts as well as unlikely to repeat. In addition, since the data was collected as the first wave of the pandemic was receding, human mobility was still limited and the society was in many ways closed, we would expect a relatively small number of random encounters.

One-off contacts. A plausible starting point, when identifying random contacts, is to look at one-off contacts, i.e., contacts that were observed on only one day in our dataset. This one-off behaviour was observed despite the fact that the involved devices were simultaneously present, that is they have registered contacts with other devices, in at least seven days. In other words, we can not explain the lack of several contacts by simply non-overlapping activity periods.

We now focus only on the three derived datasets and ignore the raw contacts since these involve many contacts that are associated with distances greater than two meters. Supplementary Table 2 shows the percentage of one-off contacts for each dataset. The percentage of one-off contacts decreased as we tightened the contact selection criteria. This is expected since the

Dataset	*naive*	*time-limited*
PC	18.6%	70.1%
RRC	19.8%	43.5%
PRC	15.6%	29.2%

Supplementary Table 3: Percentage of one-off contacts after applying simple filters for the three types of contacts: Proximity Contacts (PC), Relatively Risky Contacts (RRC) and Potentially Risky Contacts (PRC).

tighter the criteria the more likely we avoid spurious contacts. Given the state of the society at the time, we would expect a lower extent of random contacts. Accordingly, these numbers are likely to involve known close contacts, since these also were recommended to social distance. For example, friends and family who only met once during the study period. Other causes like app-related artefacts; users tendency to switch off the app when they are home and only use it when outdoors or simply people living in larger homes end up leaving their phones in separate rooms which results in a lower signal strength might have also contributed to the high fraction of one-off contacts. Hence, the percentage of random contacts will likely be greatly overestimated if we assume that all one-off contacts were random contacts.

Simple filtering. A simple approach would classify an one-off link between two devices a and b as random if and only if there exists no third node c that is connected to both a and b (i.e. a and b do not have common neighbours). This classifier, which we refer to as *naive* in the following, can result in false negatives by classifying an actual random link as non-random. For instance, two devices, which are actual close contacts, encountered a third random contact while traveling together. These devices will form a triad that would be interpreted as a sign of a non-random contact. It can also lead to false positives by classifying known contacts as random contacts. For example, friends that met only once during the study period without being in proximity to a third person simultaneously. Another naive approach for identifying random contacts is to impose a minimum time duration for accepting a contact as non-random, we refer to this classifier in the following as *time-limited*. Here, we do not take into account whether the contacts had a common neighbor or not. We set this threshold to 60 minutes, which should be enough to capture encounters with friends and neighbours for example. While this may seem plausible, such a minimum duration is a function of the type of encounter as well as the app and can vary widely.

Supplementary Table 3 shows the percentage of one-off contacts after applying the *naive* and *time-limited* filtering. The two approaches reduced the percentage of one-off contacts but with different magnitudes.

The *naive* approach returned comparable percentages of one-off contacts for the three datasets. This is unexpected given the underlying differences between the datasets with respect to minimum contact duration. The number of one-off contacts is expected to be the highest for PC and lowest for PRC. The *naive* approach is simply indicating that the likelihood of having no

**Dataset**	*Flagged*	*Not flagged*
PC	3.0%	11%
RRC	15.6%	39.5%
PRC	30.9%	55.7%

Supplementary Table 4: Percentage of contacts longer than one hour for one-off contacts that were flagged (left as one-off) and not flagged by the naive approach. These percentages are broken down by the three types of contacts: Proximity Contacts (PC), Relatively Risky Contacts (RRC) and Potentially Risky Contacts (PRC).

common neighbor is invariant to the contact duration and is consistent across the three dataset. The *time-limited* approach returned higher percentages of one-off contacts, which means that the majority of the original one-off contacts lasted less than an hour. Here, the three dataset exhibited differences that are consistent with the underlying differences between them. We further looked at the duration of the one-off contacts that were flagged by the *naive* approach in Supplementary Table 3 (i.e. those between users without common neighbours) and those which were not flagged. Supplementary Table 4 shows the percentage of contacts that were longer than one hour for both categories. Contacts between users with common neighbours were more likely to be longer. The flagged contacts involved a non-trivial percentage of long contacts, especially for PRC, which hints at the presence of false positives. The not flagged contacts included also a large fraction of short contacts, which suggests that the *naive* approach had failed in identifying a sizable fraction of random contacts (i.e. false negatives).

In summary, the two approaches yielded different results. The *naive* approach did not account for the underlying differences between the three datasets. This resulted in inferring close estimates of potential random contacts and apparently sizable fractions of both false positives and false negatives. The results of the *time-limited* approach were compatible with differences between the underlying datasets, which is expected as these differences are indirectly captured by the time threshold. However, this approach does not take into account the structure of the contact graph, which would make it vulnerable to false positives. Accordingly, a successful approach for identifying random contacts must yield results, with respect to the volume and duration of random contacts, that are consistent with the differences between the underlying dataset. Further, the results should indicate clear qualitative differences between known and random close contacts.

Machine learning classifier. To overcome these limitations, we employed a random forest binary classifier that takes into account a broader set of features[20,21]. Using a supervised classifier, we aimed to identify relationships between the features beyond the presence of common neighbours as well as to avoid imposing arbitrary thresholds on contact duration.

To train a binary classifier, we needed a training set that includes both true positives (i.e. close contacts) and true negatives (i.e. random contacts). In absence of a verified ground truth, we needed to carefully pick these two sets from the underlying data. Intuitively, *true positives*

will be users that have a high frequency of daily encounters, for instance people living or working together. We accordingly picked device pairs that had at least seven encounters, that is they met on seven different days. Identifying *true negatives* is more challenging though. One could think of picking random pairs of devices with no common neighbours. This can be a viable approach, if all devices occupy the same physical space and they can plausibly meet. However, our data covers the whole of Norway, which reduces this plausibility and renders the above approach inadequate. We instead reverted to the contact pattern to discern potential true negatives. Assume that devices a and b have met each others frequently, while device c has more than two encounters with device a but has never been in the proximity of device b. The higher the frequency at which a and b meet, the less likely that c has ever been in the proximity of b. In this example, a and b could be family members and a and c coworkers, so b and c are true negatives that never met during the data collection period despite the presence of common neighbours. Note that we did not require that a and c have only one common neighbour, since that would directly influence the definition of random contacts. We expected nevertheless true negatives to share fewer neighbours if any, which serves as a reasonable test to verify the identified true negatives. To confirm this, we plotted the density of the number of common neighbours for both true positives (TP) and true negatives (TN) (see Supplementary Figure 4). The plot matched our expectations. TNs shared only one neighbour in most cases.

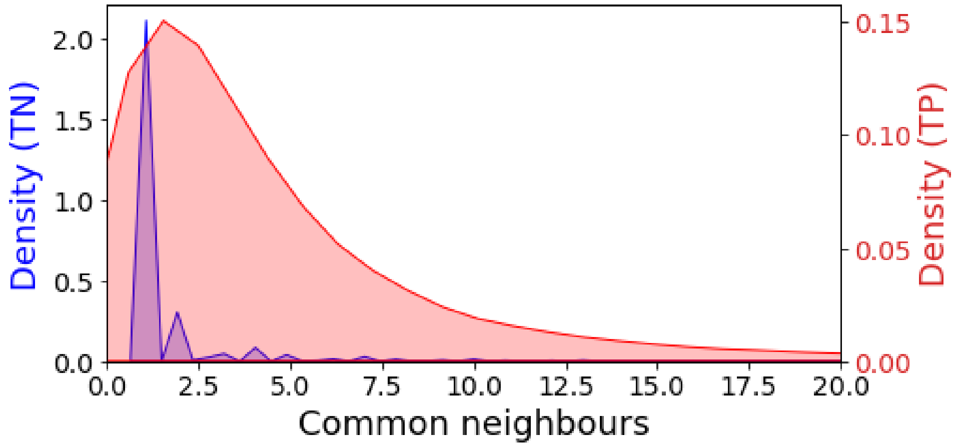

Supplementary Figure 4: The density of the number of common neighbours for both the inferred true positives and true negatives. TNs have clearly fewer common neighbours.

Features. We trained our model using a total of nine features, which were meant to capture the quality of information we have on a pair of devices, their connectivity as well as the topological commonalities between them.

1. device visibility and overlap features: for a pair of devices a and b, we collected the number of days each device was active, i.e., either discovered or was discovered by another device. We also collected the number of days both devices were active, which we refer

to as availability overlap. These features were meant to control for the effect of devices' measurement coverage on the likelihood they discover each other repeatedly.

2. Graph and topological commonalities features: for a pair of devices, we collected each device degree (i.e. the total number of unique contacts it recorded) and the number of common neighbors. We further computed two features to capture the similarity in connectivity: Jaccard's index [22] and Adamic/adar (AA) index[23]. For a pair of devices with a set of neighbours $N(a)$ and $N(b)$, Jaccard's index is given by $\frac{|N(a) \cap N(b)|}{|N(a) \cup N(b)|}$, which is basically the fraction of common neighbours. The AA index is defined as the summation of the inverse logarithmic degree centrality of the neighbours shared by pair, it is given by $\sum_{c \in N(a) \cup N(b)} \frac{1}{log|N(c)|}$. Essentially, the AA index is higher if devices tend to commonly connect to low degree devices than high degree ones. Both metrics have been used before to predict missing links in social networks[24].

3. device type features: for each pair of devices, we input their device type(s), which is either Android or iOS.

Note that the aforementioned features do not include contact duration since we do not have a clear mapping between contact duration and type. Also, these features can only help classifying one-off contacts with common neighbours, that is the difference between the initial one-off contacts in Supplementary Table 2 and those identified by the *naive* approach in Supplementary Table 3. For example, it will classify over 70% of the one-off contacts in the PRC dataset. Classifying one-off contacts without common neighbours requires extra features about contexts of contacts that do not exist in our dataset. We argue that this is not a major limitation because the majority of one-off contacts were between users with common neighbours. More specifically, the share of one-off contacts between users with common neighbours is 75.8%, 69.2 and 72.8% for PC, RRC and PRC respectively. Also assuming that one-off contacts without common neighbours are not random will give a conservative estimate of the share of random contacts.

Classifier. We train a random forest classifier with 20 trees, gini criterion, a maximum tree depth of 8, a minimum number of samples required to split an internal node of 2 and a minimum number of samples required to be at a leaf node of one. The values of these hyperparameters were selected after conducting an exhaustive grid search.

classification accuracy. We fitted three models for the PC, RRC and PRC dataset, respectively. We performed k-fold cross validation for each model to verify that our models can generalize. The three models exhibited a high level of accuracy as follows: PC (84%), RRC (88%) and PRC (89%). Supplementary Tables 5, 6 and 7 present the confusion matrices for the three datasets.

All three models exhibited a higher accuracy when classifying close contacts. The performance, however, slightly degraded when classifying random contacts, where a higher fraction of them was classified as close contacts. Hence, these models are conservative when it comes

Predicted contact / Real contact	Random	Close
Random	80.5%	19.5%
Close	12.2%	87.8%

Supplementary Table 5: Proximity Contacts (PC) confusion matrix.

Predicted contact / Real contact	Random	Close
Random	86.0%	14.0%
Close	9.6%	90.4%

Supplementary Table 6: Relatively Risky Contacts (RRC) confusion matrix.

Predicted contact / Real contact	Random	Close
Random	86.7%	13.3%
Close	8.3%	91.7%

Supplementary Table 7: Potentially Risky Contacts (PRC) confusion matrix.

Feature	Gini importance
AA's index	0.398
Number of common neighbours	0.162
Device A total days with measurements	0.122
Device B total days with measurements	0.116
Jaccard's index	0.102
Total overlapping days with measurements	0.079
Device A's degree	0.007
Device B's degree	0.007
Type of phones	0.007

Supplementary Table 8: Features importance.

to flagging an encounter as a random contact. This is a desirable property, because this way the model will not lead to overestimating the benefits of digital contact tracing. We also note that the accuracy of the model improved as the definition of contacts became stricter. The stricter the definition the less likely the contact is a false positive. Consequently, classifying these contacts will be less error prone.

Feature importance. We also investigated the role of different features and their contribution to the model's accuracy. To this end, we use the Gini importance or the Mean Decrease in Impurity measure, which counts the fraction of times a feature is used in determining how to split the classification tree.

Supplementary Table 8 shows the Gini's importance for all features. AA's index and the number of common numbers were important to more than half of the decisions. Then followed by the features that describe the quality of the measurement data. Accordingly, the model has learned to classify contacts depending mainly on the features that capture topological commonalities between devices. Note that although the number of common neighbours and Jaccard's index capture some of the topological similarity aspects that are captured by AA-index, the latter is more important for discriminating contacts. This could be attributed to the fact that AA-index refines the neighbourhood comparison beyond simple counting by considering structural similarities.

Inferred random contacts. The random forest model classifies a non-trivial fraction of one-off contacts as close contacts, which we summarize in Supplementary Table 9. Only 11% of PRC contacts were classified as random. If we consider only the contacts that we could classify (i.e. ignore one-off contacts between pairs without common neighbours), the percentage of random contacts increased slightly to 40.7%, 18.3% and 13.1% for PC, RRC and PRC, respectively. The inferred fractions of random contacts are similar to numbers suggested by previous studies of social contacts as well as reports on untraceable Covid-19 infections[25,26]. Supplementary Figures 5 and 6 show how the fraction of random contacts has evolved in period for the PC and RRC datasets, respectively. Our estimates of random contacts are conservative, since all

Dataset	Random contacts (%)
PC	33.3%
RRC	14.5%
PRC	11.0%

Supplementary Table 9: Percentage of one-off contacts after applying the random forest classifier. These percentages are broken down by the three types of contacts: Proximity Contacts (PC), Relatively Risky Contacts (RRC) and Potentially Risky Contacts (PRC).

one-off contacts between users without common neighbours are assumed not to be random contacts. Now, if we assume that all such contacts lasting less than an hour were random, the fraction of random contacts would increase to 20.8%, 31.2% and 51.4% for PRC, RRC and PC, respectively.

A limitation of our approach is that the underlying contact graph is incomplete, that it does not include the entire adult population of Norway. This incompleteness can lead to classifying close contacts as random contacts. We attempted to minimize the impact of this by including users that sent data on at least seven days as well as pair of devices that were sending data on at least the same seven days. We also considered all one-off contacts between users without common neighbours as close since we did not have features that captured the contexts of these contacts. We could not gauge the impact of graph incompleteness on our inference because we lacked ground truth. However, the qualitative differences between random contacts and close contacts in terms of contact duration and number of contacts of each type (see Figure 3 in the main text), suggest that we are discriminating contacts of different underlying characteristics.

Supplementary Note 6: Highly connected users

We investigated the users with a total number of contacts of 50 or more. Approximately 4% of all users fall into this category. Overall, we have 19656 such users, 12410 of them had 10% or more random contacts. Further, 4812 and 1538 of these highly connected users had at least 20% and 30% random contacts, respectively. This indicates that highly connected users tend to mostly connect to frequent contacts. However, they are also associated with a non-trivial fraction of random contacts, which may result in large outbreaks in case a high connected user was infected. Breaking down the contacts involving highly connected users per day of the week shows interesting trends (see Supplementary Figure 7). The fraction of such contacts that corresponds to encounters with known (frequent) contacts peaked during the week days and dropped at weekend. The encounters with unknown (random) contacts also peaked in the middle of the week, but it exhibited a slight uptick on Sunday. The pronounced activity during the week indicates that these highly connected users are probably individuals with jobs that put them in proximity of many other people. The small uptick on Sunday for unknown contacts may hint at social gatherings. Taking this one step further, we estimated for each highly connected user

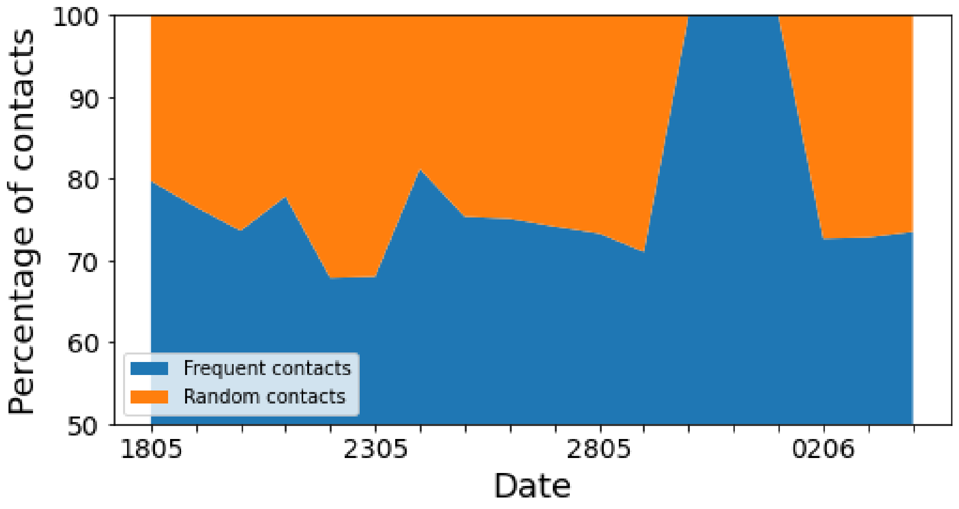

Supplementary Figure 5: The timeseries of the fraction of random contacts for the Proximity Contacts (PC) dataset.

the fraction of known contacts as well as the fraction of unknown contacts that were also highly connected. Our results showed non-trivial positive assortativity (see Supplementary Figure 8). Both distributions peaked around 0.3. Interestingly, the distribution of known contacts exhibited a mode at 1.0, which indicates the presence of communities of highly connected users. This non-trivial positive assortativity regardless of the contact type hints at different potential explanations for the observed high connectedness.

Supplementary Note 7: The effectiveness of exposure notification system-based contact tracing apps

The exposure notification system (ENS) developed by Apple and Google has emerged as the de-facto standard for digital contact tracing[27]. Currently, 28 countries and 19 US states US have already rolled out ENS-based apps[28]. Key to the success of the ENS is the built-in privacy preservation and expected superior performance.

The built-in privacy preservation, however, makes the task of precisely assessing apps effectiveness impossible. If an app user tested positive for Covid-19, he or she is handed a one-time code to register the test results in the app, which in turn uploads a set of keys, one key per day for at most the last 14 days, that identifies the index case to a central server. Other app users download periodically all uploaded keys and match them with all saved encounters to determine whether they have been in proximity of an index case. In this process, health authorities hand out the one-time codes and configure the ENS to define close contacts.

Quantifying the effectiveness of ENS-based apps is a multi-step process. The first step verifies the accuracy of inferred contacts. The accuracy depends on the suitability of BLE

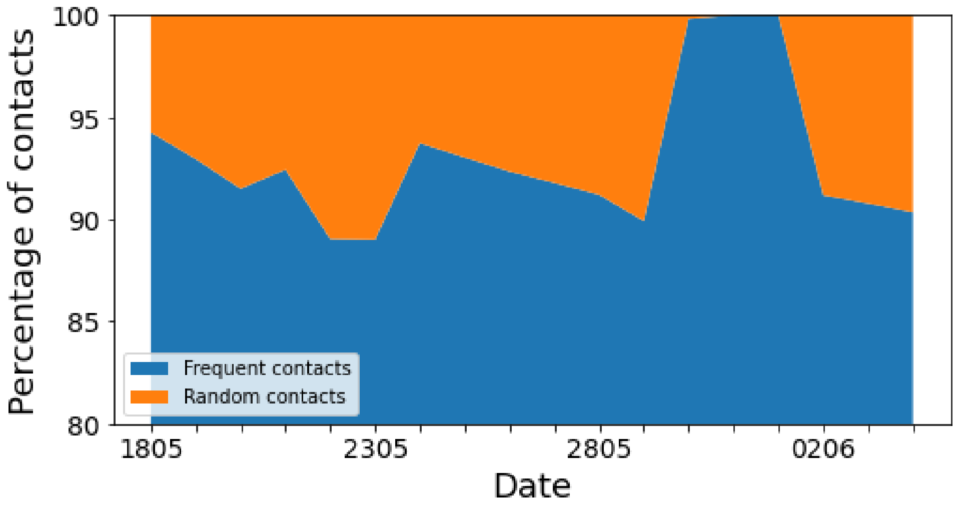

Supplementary Figure 6: The timeseries of the fraction of random contacts for the Relatively Risky Contacts (RRC) dataset.

signal attenuation in inferring distances and the configuration of the ENS. Several efforts have examined these two aspects and showed that ENS, like any other BLE-based distance estimation system, can have unpredictable performance due to wireless propagation artefacts[12,13]. They also showed that ENS configurations in use by many countries tend to miss real contacts. As a response many countries are continuously monitoring their configurations. Note that all these efforts were limited to a small number of phones, usually less than hundred. The second step tracks the adoption rate of the app, that is the fraction of population that have installed the app, as well as the fraction of index cases that have the app installed. Unfortunately, both numbers can be at best approximated. Health authorities can collect statistics from app stores about how many users have downloaded the app, but these app stores do not track uninstalls. They can also instrument the apps, by for example asking each app to connect to a central server to check for configuration changes x times per day[29]. There is no automated way to check whether an index case has the app. Manual contact tracers or laboratories issue the case a one-time code to report the diagnosis to the app, which in turn triggers the upload of exposure keys. This has inherent limitations since the case can choose not to report having the app. Furthermore, the index case can also choose not to report the diagnosis. Health authorities can track both numbers since they issue the one-time codes and later verify them. The last step measures the epidemiological benefit by tracking false positives and false negatives in comparison with manual contact tracing. These can be estimated by surveying individuals tested for Covid-19 about the use of the app and whether they were notified through it. The test results can then reveal whether digital contact tracing is identifying epidemiologically risky contacts.

The available followup data on deployed ENS-based apps is limited. We used publicly available statistics about the German (Corona Warn) and Swiss (SwissCovid) apps to gauge

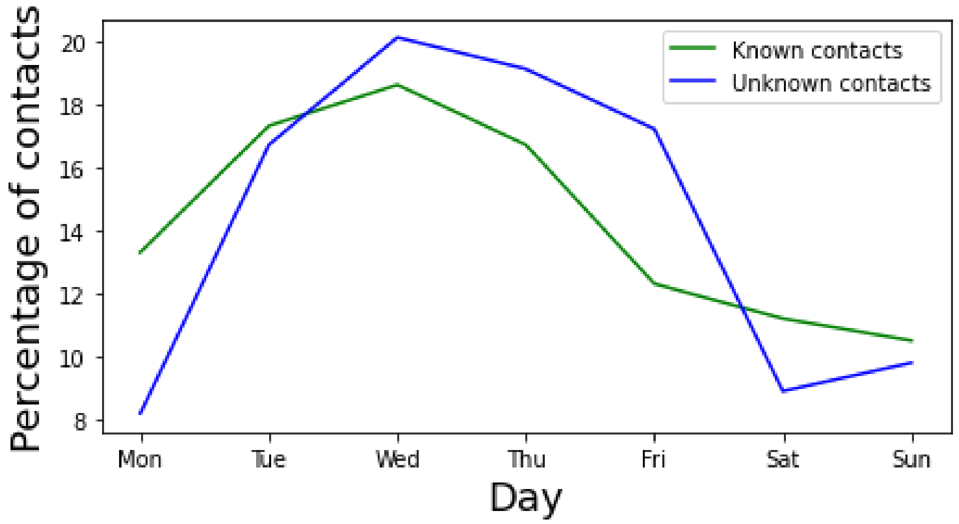

Supplementary Figure 7: The percentage of contacts involving highly connected neighbours per day of week.

their effectiveness[30,31]. These two apps were rolled out in June 2020. In particular, we used the published numbers about diagnosis upload via the app, the app downloads and the number of inquiries to the health system following the reception of an exposure notification. We analyzed 3 months worth of data for Corona Warn and over 4 months for SwissCovid.

Supplementary Figures 9 and 10 present three key measures for both apps. The app coverage, that the fraction of index cases with the app installed and have requested a one-time code, follows closely the apps uptake ratio. Further, the coverage remains at the same level as the number of cases surges. Hence, the app uptake seems to be a good proxy for gauging the case coverage. Each uploaded one-time code generates at least one call to the app hotline, a phone number that is only revealed when a user is notified of a potential risky contact, which is an indirect indicator of the effect of the app. Note that Corona Warn makes available both the number of people received one-time codes and those decided to enter them, while SwissCovid makes only the latter available. Between September 2020 and January 2021, only 55% of index cases uploaded the received one-time codes to Corona Warn. Earlier analysis reported a higher fraction, 66.2%, for SwissCovid[32]. Both numbers are low essentially halving the effectiveness of the apps.

We also looked at average app coverage in comparison with adoption rate for Denmark based on the publicly available numbers[33]. The Danish app was rolled out in June and it has been, by January 3rd 2021, downloaded by 35% of the population. The average coverage of the Danish app is at 29% compared to 14% and 11% for Switzerland and Germany, respectively. About 80% of index cases in Denmark, with app installed, have opted to submit their test diagnosis.

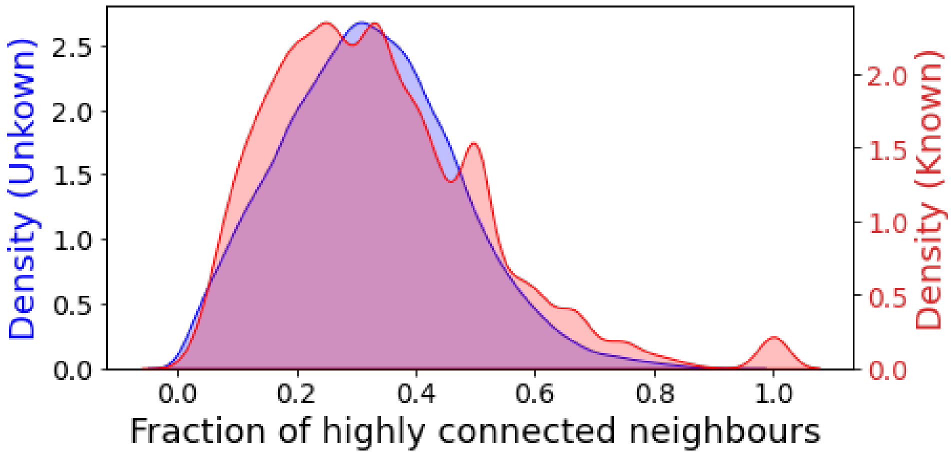

Supplementary Figure 8: The distribution of both known and unknown neighbours of a highly connected user that are also highly connected.

Under 18		Over 18	
Household	3.02	Household	1.90
School	5.54	Work	5.94
Other	1.47	Other	3.74

Supplementary Table 10: Mean number of contacts in each catoegory, calculated from the supplementary data provided with[34].

Supplementary Note 8: Estimating the efficacy of manual contact tracing

An attempt to estimate the number of traceable contacts is provided by Kucharski et al.[34]. The study is based on the BBC Pandemic dataset, which contains self-reported contacts in the form of a face-to-face conversation ($>$ 3 words) or a contact involving physical touch. Contacts were grouped into household contacts, work, school, and other. For each contact, responders also reported whether they knew this person (i.e. if had they met before). Kucharski et al. classified a contact as non-traceable if the two persons had not met before, and arrived at the following proportion of traceable contacts: household contacts 100%, school contacts 90%, work 71%, and other contacts 52%. These proportions were combined with the mean number of contacts in each category, listed in Supplementary Table 10 to estimate the total number of traceable and non-traceable contacts. For instance, a person over 18 will have, on average, 7.62 traceable contacts and 5.26 non-traceable contacts per day.

There are several limitations in the use of the BBC pandemic dataset for estimating traceable contacts. Since the data is based on self-reporting, it only considers contacts in the form of conversations and physical contact. Other contacts, such as being in close proximity on public transportation, are not included. Furthermore, there is (to our knowledge) no threshold on the

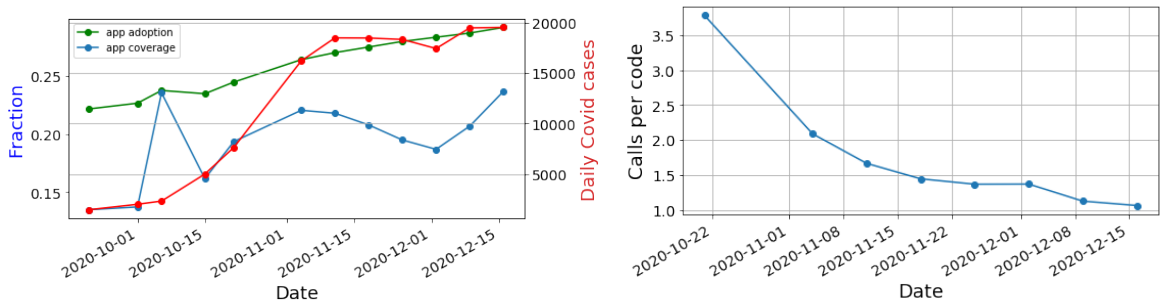

Supplementary Figure 9: (**Left**)Left Y-axis: the adoption rate of Corona Warn measured as the fraction of the German population that downloaded the app and the app coverage measured as the fraction of index cases that requested one-time codes. The right Y-axis shows the daily confirmed Covid cases. (**Right**) the average number of calls to the Corona Warn hotline per each issued one-time code

duration of a contact. A very short exchange of words, lasting less than a minute, will still be counted as a contact, while manual contact tracing typically uses ten minutes as a guideline threshold.

Authorities in many countries publish reports on infected cases and the source of infection, which provides an alternative data source for estimating the efficacy of manual contact tracing. For instance the local health authorities in Oslo, Norway reported 423 cases from October 19 to October 25, 2020. For 96 of these cases, (23%), the source and location of infection were not known. From October 26 to November 2, 179 (25%) out of 716 reported cases had an unknown source[26].

The impact of tracing random contacts on the pandemic. If we assume, for instance, 60% app uptake in the population, we observe from Figure 2 in the main text that the efficacy of the app tracing is approximately 30%. We may further assume that the app is used as a supplement to manual contact tracing, and that its main purpose is to trace contacts that are not traceable by manual tracing. Based on the BBC pandemic dataset, Kucharski et al[34] estimated that 41% of contacts were non-traceable. This estimate gives maximum efficacy of 59% and adding digital tracing with 30% efficacy will increase the overall tracing efficacy to 71%. As previously demonstrated by Ferretti et al[35], a difference of this magnitude can easily mean the difference between a controlled pandemic and an exponential growth of cases.

Supplementary Note 9: Modeling the effect on the pandemic spread

We have used the model of Ferretti et al[35] to quantify the potential effect of the digital contact tracing on the pandemic spread. In[35] the model was used to quantify the effect of efficacy and delay in isolation of infected individuals and tracing of their contacts, while for the present study our primary interest is the potential effect of app uptake. To quantify this effect, we cal-

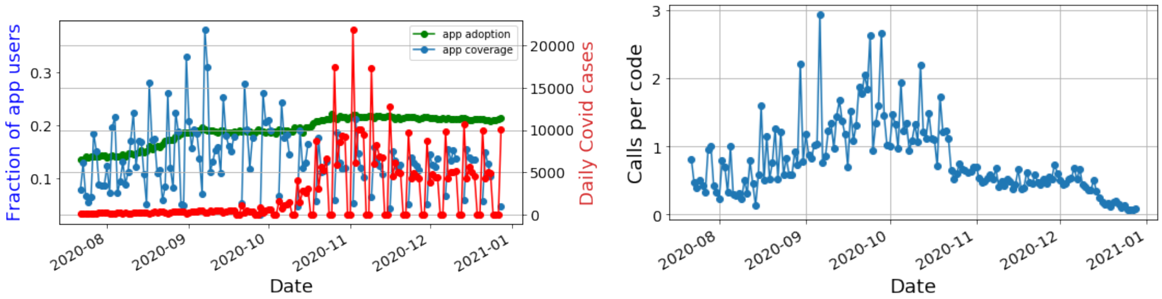

Supplementary Figure 10: (**Left**)Left Y-axis: the adoption rate of SwissCovid measured as the fraction of the Swiss population that downloaded the app and the app coverage measured as the fraction of index cases that requested one-time codes. The right Y-axis shows the daily confirmed Covid cases. (**Right**) the average number of calls to the SwissCovid hotline per each issued one-time code

Parameter	Value(s)	Source
Reproduction number (R0)	1.5, 2.7	[36]
Efficacy of contact tracing	0-100%	Calculated from Eq. 5 in the main text
Efficacy of case isolation	50%, 70%, 90%	[34, 35, 37, 38]
Delay of tracing and isolation	4 hrs, 24 hrs, 48 hrs	[35]

Supplementary Table 11: Parameters that are varied in our application of the model from[35] to assess the effect of app uptake on the pandemic spread.

culated the tracing efficacy as a function of app uptake for the two operating systems, given by Eq. 5 in the main text , and used these numbers as input to the model. Although efficacy of self-isolation and delays in quarantining and isolation are not a a primary focus of the present work, we used multiple values for these parameters to investigate their potential impact on results and conclusions. The model parameters that were varied in our calculations are specified in Supplementary Table 11. All other parameters were fixed at their default values specified in[35].

Supplementary Figure 11 shows the growth rate as a function of app uptake for $R0 = 1.5$, for case isolation efficacy of 50% (top), 70% (middle) and 90% (bottom), and for delays of 4, 24, and 48 hours, respectively, from left to right. The black line shows the limit $r = 0$, which marks the difference between exponential growth and a decaying pandemic. As previously demonstrated in[35], both the delay and isolation efficacy impact the overall effectiveness significantly. For a delay of 48 hours and a isolation efficacy of 50% (upper right corner) the app uptake must be around 75% to control the pandemic spread. Assuming a more realistic, yet still conservative, isolation efficacy of 70%, in combination of a four hour delay, around 40% app uptake is sufficient. The lower right panel shows that 90% effective isolation of cases with four hours delay should be sufficient to contain the pandemic, without any tracing of contacts.

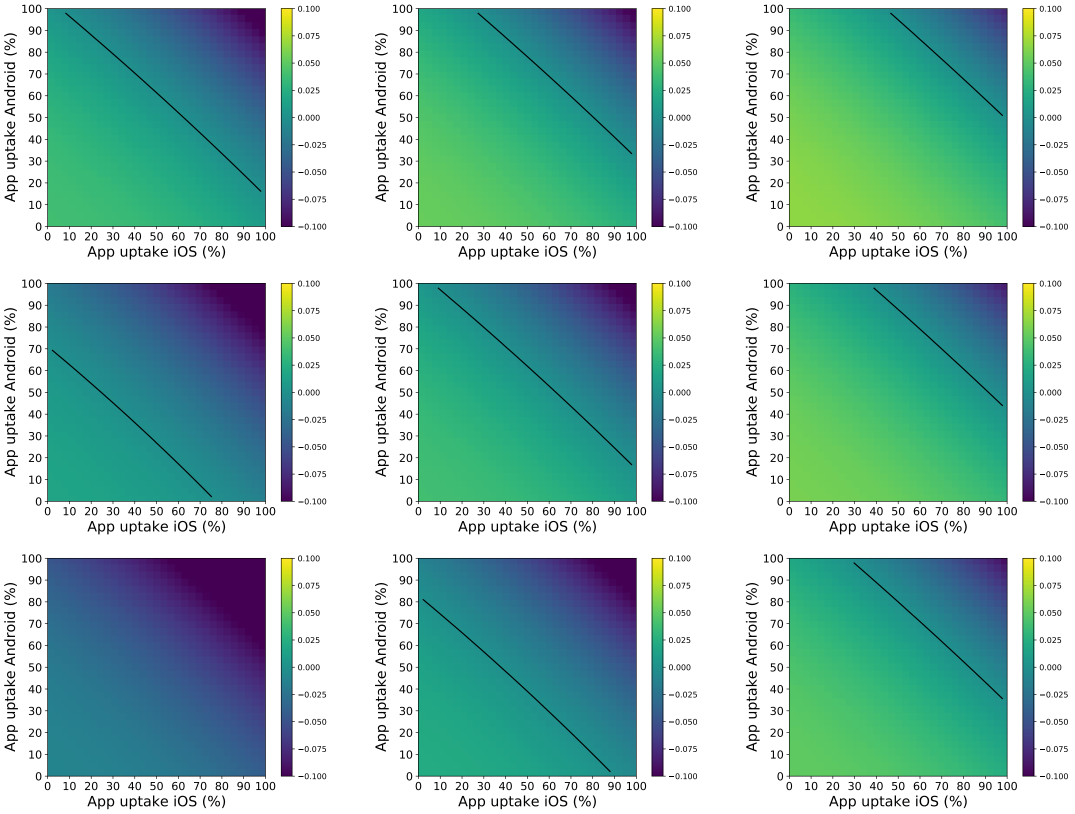

Supplementary Figure 11: The figures show the exponential growth rate r as a function of app uptake, for different choices of model parameters. The reproduction number $R0 = 1.5$ for all plots. For the top row the efficacy of isolating infected cases is set to 50%, middle row 70%, and bottom row 90%. From left to right shows a delay of quarantining and isolation of 4, 24, and 48 hours, respectively.

This result is in line with the model results shown in[35], and indicates that at this moderate reproduction number the pandemic cannot be driven solely by infections from pre-symptomatic individuals. Supplementary Figure 12 shows the same results as Supplementary Figure 11, but for $R0 = 2.7$. We see that for this reproduction number it is not realistic to control the pandemic based on on digital contact tracing alone, since even for the highest isolation efficacy and lowest delay (lower left corner) the necessary uptake is around 90% .

Supplementary Note 10: Legality of the data set

The suspension of Smittestopp was decided in June 2020 after the Norwegian Data Protection Authority issued a warning that the app could be considered illegal under GDPR. There were two main reasons. First, the infection rate in Norway had dropped to almost zero, so the

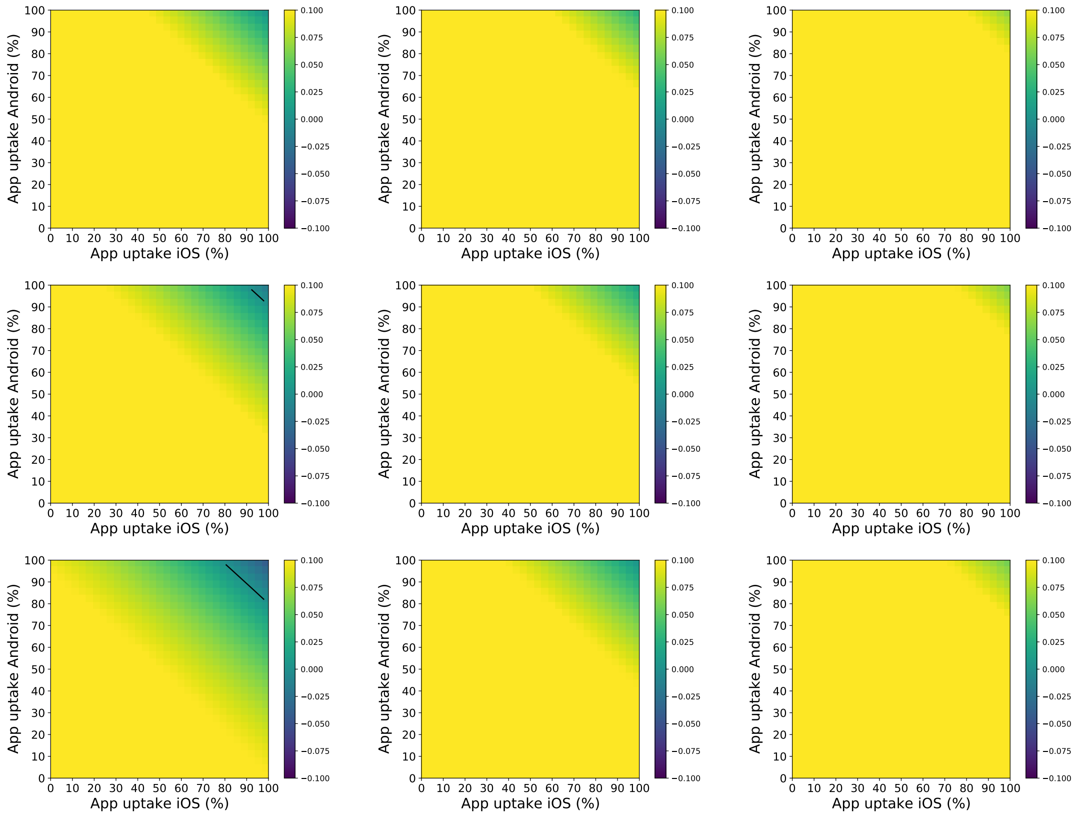

Supplementary Figure 12: The figures show the exponential growth rate r as a function of app uptake, for different choices of model parameters. The reproduction number $R0$ is set to 2.7 for all plots. For the top row the efficacy of isolating infected cases is set to 50%, in the middle row 70%, and in the bottom row 90%. From left to right shows a delay of quarantining and isolation of 4, 24, and 48 hours, respectively.

collection of data could no longer be considered proportional. Second, Apple and Google had launched a technology for contact tracing that appeared to have high efficacy without the need for central storage of data. The considerations on data minimization therefore changed when Switzerland as the first country was testing an app based on this new technology in late May. Smittestopp was soon discontinued, and it was eventually decided to build a new app based on the technology from Apple and Google. Since the app was shut down, the Norwegian Data Protection Authority never concluded on their warning.

We received the necessary consent from the Norwgian Institute of Public Health to use the data-set for research purposes. Still, the privacy consideration of Smittestopp was subject to a fierce public debate, thus any use of data from this app had to be done with great care. We have therefore collected separate advice from the Norwegian Centre for Research Data[2] (NSD) on the

[2]https://www.nsd.no/en

risk of re-identification based on our data-set, and from the Norwegian Law-firm Wiersholm[3] on the legality of the data-set.

The law firm Wiersholm concludes that the data-set can be legally used for research purposes if there does not exist additional information that would make it possible to re-identify persons under the assumption that all reasonable means for re-identification is used. NSD writes that it is hard to imagine how a motivated intruder can successfully re-identify anyone, given that the data in the set is not part of any other registries. Simula Research Laboratory therefore conclude that use of the data-set for research purposes is legal.

As an extra level of caution, Wiersholm advises us not to make the data-set public, but rather share it with other researchers under agreements of non-disclosure and usage limitation. Our treatment of the data-set is in line with this advice.

References

[1] N. I. of Public Health, Smittestopp – ny app fra folkehelseinstituttet, https://www.fhi.no/nyheter/2020/ny-app-fra-folkehelseinstituttet/ (accessed January 26, 2021).

[2] Datatilsynet, Midlertidig stans av appen smittestopp, https://www.datatilsynet.no/aktuelt/aktuelle-nyheter-2020/midlertidig-stans-av-appen-smittestopp/ (accessed January 2021).

[3] N. I. of Public Health, Ny smittestopp-app klar for nedlasting, https://www.fhi.no/nyheter/2020/ny-smittestopp-app-klar-for-nedlasting/ (accessed January 26, 2021).

[4] Smittestopp, ”https://github.com/smittestopp” (accessed February 11, 2021).

[5] Bluetooth, SIG, Inc., Bluetooth Specification Version 4.2.

[6] A. van Rossum, Smartphone localization, https://github.com/crownstone/bluenet-ios-basic-localization/blob/master/BROADCASTING_AS_BEACON.md (accessed January 26, 2021).

[7] D. G. Young, Hacking The Overflow Area, http://www.davidgyoungtech.com/2020/05/07/hacking-the-overflow-area (accessed January 21, 2021).

[8] G. T. Agency, 6 things about OpenTrace, the open-source code published by the TraceTogether team, https://www.tech.gov.sg/media/technews/six-things-about-opentrace#6-last-but-not-least-an-extra-step-for-ios-users (accessed January 26, 2021).

[9] Apple Developer Documentation, iBeacon, https://developer.apple.com/ibeacon/ (accessed January 26, 2021).

[10] Apple Developer Documentation, Core Location, https://developer.apple.com/documentation/corelocation/ (accessed January 26, 2021).

[3]https://www.wiersholm.no/en

[11] S. H. Standard, *US Doc/NIST* (2002).

[12] Sammenligning av alternative løsninger for digital smittesporing, Simula Research Laboratory, 2020, "https://www.simula.no/sites/default/files/sammenligning_alternative_digital_smittesporing.pdf" (accessed January 26, 2021).

[13] D. J. Leith, S. Farrell, *Plos one* **15**, e0239943 (2020).

[14] T. Lovett, *et al.*, *arXiv preprint arXiv:2007.05057* (2020).

[15] D. J. Leith, S. Farrell, *ACM SIGCOMM Computer Communication Review* **50**, 66 (2020).

[16] Z. J. Madewell, Y. Yang, I. M. Longini, M. E. Halloran, N. E. Dean, *JAMA network open* **3**, e2031756 (2020).

[17] W. Li, *et al.*, *Clinical Infectious Diseases* **71**, 1943 (2020).

[18] M. Hu, *et al.*, *Clinical Infectious Diseases* (2020).

[19] H.-Y. Cheng, *et al.*, *JAMA internal medicine* (2020).

[20] T. K. Ho, *Proceedings of 3rd international conference on document analysis and recognition* (IEEE, 1995), vol. 1, pp. 278–282.

[21] G. James, D. Witten, T. Hastie, R. Tibshirani, *An introduction to statistical learning*, vol. 112 (Springer, 2013).

[22] L. Hamers, *et al.*, *Information Processing and Management* **25**, 315 (1989).

[23] L. A. Adamic, E. Adar, *Social networks* **25**, 211 (2003).

[24] T. Zhou, L. Lü, Y.-C. Zhang, *The European Physical Journal B* **71**, 623 (2009).

[25] J. Mossong, *et al.*, *PLoS Med* **5**, e74 (2008).

[26] Statusrapport covid19 (2020). Report on Covid 19 in the Municipality of Oslo, published Nov 3, 2020 (in Norwegian).

[27] Apple and Google, Apple and google partner on covid-19 contact tracing technology, https://www.apple.com/newsroom/2020/04/apple-and-google-partner-on-covid-19-contact-tracing-technology/, accessed January 8, 2021 (2020).

[28] Exposure Notification, https://en.wikipedia.org/wiki/Exposure_Notification (accessed January 5, 2021).

[29] Calculation methods for estimating the number of active SwissCovid apps, https://www.experimental.bfs.admin.ch/expstat/en/home/innovative-methods/swisscovid-app-monitoring.assetdetail.13667538.html (accessed February 5, 2021).

[30] Corona-warn-app, https://www.coronawarn.app/en/ (accessed January, 26 2021).

[31] Swisscovid, https://www.bag.admin.ch/bag/en/home/krankheiten/ausbrueche-epidemien-pandemien/aktuelle-ausbrueche-epidemien/novel-cov/swisscovid-app-und-contact-tracing.html (accessed January, 26 2021).

[32] M. Salathé, *et al.*, *medRxiv* (2020).

[33] Driftsstatus, https://smittestop.dk/status/ ((accessed January 5, 2021)).

[34] A. Kucharski, *et al.*, *Lancet Infect Dis* pp. 30457–6 (2020).

[35] L. Ferretti, *et al.*, *Science* **368** (2020).

[36] https://www.fhi.no/en/publ/2020/weekly-reports-for-coronavirus-og-covid-19.

[37] J. Almagor, S. Picascia, *Scientific reports* **10**, 1 (2020).

[38] J. Hellewell, *et al.*, *The Lancet Global Health* **8**, e488 (2020).
