# Supplementary material for: Nationwide rollout reveals efficacy of epidemic control through digital contact tracing
Source: Nat Commun. 2021 Oct 11;12:5918. doi: 10.1038/s41467-021-26144-8 (PMC8505561; doi:10.1038/s41467-021-26144-8)
Supplement: Supplementary file 2 — Reporting Summary [file 41467_2021_26144_MOESM2_ESM.pdf]

Corresponding author(s): Ahmed Elmokashfi

Last updated by author(s): Sep 17, 2021

Reporting Summary

Nature Research wishes to improve the reproducibility of the work that we publish. This form provides structure for consistency and transparency in reporting. For further information on Nature Research policies, see our Editorial Policies and the Editorial Policy Checklist.

Statistics

For all statistical analyses, confirm that the following items are present in the figure legend, table legend, main text, or Methods section.

n/a	Confirmed	
☐	☒	The exact sample size (n) for each experimental group/condition, given as a discrete number and unit of measurement
☐	☒	A statement on whether measurements were taken from distinct samples or whether the same sample was measured repeatedly
☐	☒	The statistical test(s) used AND whether they are one- or two-sided<br>*Only common tests should be described solely by name; describe more complex techniques in the Methods section.*
☒	☐	A description of all covariates tested
☒	☐	A description of any assumptions or corrections, such as tests of normality and adjustment for multiple comparisons
☒	☐	A full description of the statistical parameters including central tendency (e.g. means) or other basic estimates (e.g. regression coefficient) AND variation (e.g. standard deviation) or associated estimates of uncertainty (e.g. confidence intervals)
☒	☐	For null hypothesis testing, the test statistic (e.g. F, t, r) with confidence intervals, effect sizes, degrees of freedom and P value noted<br>*Give P values as exact values whenever suitable.*
☒	☐	For Bayesian analysis, information on the choice of priors and Markov chain Monte Carlo settings
☐	☒	For hierarchical and complex designs, identification of the appropriate level for tests and full reporting of outcomes
☒	☐	Estimates of effect sizes (e.g. Cohen's d, Pearson's r), indicating how they were calculated

Our web collection on statistics for biologists contains articles on many of the points above.

Software and code

Policy information about availability of computer code

Data collection	The dataset was collected by the Smittestopp project using database queries against Azure cloud
Data analysis	The data analysis script were developed using Python version 3.8.10, R version 4.0.3 and Perl version 5.30.0. No commercial tool was used.

For manuscripts utilizing custom algorithms or software that are central to the research but not yet described in published literature, software must be made available to editors and reviewers. We strongly encourage code deposition in a community repository (e.g. GitHub). See the Nature Research guidelines for submitting code & software for further information.

Data

Policy information about availability of data

All manuscripts must include a data availability statement. This statement should provide the following information, where applicable:

- Accession codes, unique identifiers, or web links for publicly available datasets
- A list of figures that have associated raw data
- A description of any restrictions on data availability

The data used for calculating the detection probabilities between different pairs of architectures in this study have been deposited in a public Github repository at https://github.com/sundnes/smittestoppdatamodel. The contact data set is available to research institutions and for research purposes upon request to Simula Research Laboratory as an extra level of caution due to privacy concerns (please refer to Ethical consideration and Supplementary Note 10 for extra details). Requests will be authorized by Simula's management. Correspondence and requests for materials should be addressed to Ahmed Elmokashfi or post@simula.no. The data for assessing the impact of uptake on pandemic progression and analysis of the ENS are available in the same public Github repository.

Field-specific reporting

Please select the one below that is the best fit for your research. If you are not sure, read the appropriate sections before making your selection.

- [x] Life sciences
- [ ] Behavioural & social sciences
- [ ] Ecological, evolutionary & environmental sciences

For a reference copy of the document with all sections, see nature.com/documents/nr-reporting-summary-flat.pdf

Life sciences study design

All studies must disclose on these points even when the disclosure is negative.

Sample size	We used all the aggregated contact data that was collected by Smittestopp for the period between May 18th and June 4th 2020
Data exclusions	We excluded the data that was collected prior to May 18th, because Smittestopp suffered artifacts that would lead to underestimating the occurrence of contact events. We describe these artifacts in the supplementary information (Supplementary Note 1.1).
Replication	The results can be confirmed by analyzing the contacts dataset.
Randomization	Our data set involves all the contacts that Smittestopp identified, we do not have ground truth on the type and context of these contacts. Accordingly, we could not divide them into known subgroups and subject them to different treatments. Furthermore, we did not need to control for covariates, since our study did not directly model the observed data. The machine learning model we developed leveraged features that are inherently uncorrelated. Finally, we used k-fold cross validation to ensure that our machine learning model is not overfitted. In general, the goal of our study is to quantifying the effectiveness of digital contact tracing using the entire dataset.
Blinding	Since our task was focused in analyzing and characterizing the entire measurement data set, blinding was not relevant. More specifically, we could not perform any intervention since the app was already suspended at the time of the study.

Reporting for specific materials, systems and methods

We require information from authors about some types of materials, experimental systems and methods used in many studies. Here, indicate whether each material, system or method listed is relevant to your study. If you are not sure if a list item applies to your research, read the appropriate section before selecting a response.

Materials & experimental systems

n/a	Involved in the study	
x		Antibodies
x		Eukaryotic cell lines
x		Palaeontology and archaeology
x		Animals and other organisms
x		Human research participants
x		Clinical data
x		Dual use research of concern

Methods

n/a	Involved in the study	
x		ChIP-seq
x		Flow cytometry
x		MRI-based neuroimaging
